# Subjective experience, self-efficacy, and motivation of professional football referees during the COVID-19 pandemic

**DOI:** 10.1057/s41599-023-01720-z

**Published:** 2023-05-08

**Authors:** Fabio Richlan, J. Lukas Thürmer, Jeremias Braid, Patrick Kastner, Michael Christian Leitner

**Affiliations:** 1 Centre for Cognitive Neuroscience, Paris-Lodron-University, Salzburg, Austria; 2 Department of Psychology, Paris-Lodron-University, Salzburg, Austria; 3 Department of Psychology, Ludwig-Maximilians-University, Munich, Germany; 4 Salzburg University of Applied Sciences, Salzburg, Austria

**Keywords:** Psychology, Cultural and media studies

## Abstract

The present multi-study article investigates the subjective experience of professional football (a.k.a. soccer) referees and players during the COVID-19 pandemic and the so-called ghost games (i.e., games without supporters). Referees from the Austrian Football Association completed questionnaires inquiring about self-efficacy, motivation, and general personal observations and perceptions (e.g., arousal or confidence). In addition, two players and one referee in the Austrian Football Bundesliga were interviewed retrospectively regarding their subjective experience during ghost games and the effects of emotions on behavior and performance using semi-structured, video-taped interviews. Results of the referee survey indicate that the most profound differences between regular games and ghost games lie in the domain of intrinsic motivation and multiple aspects of subjective experience. Specifically, the experience in ghost games compared with regular games was reported by referees as being significantly less motivating, less excited/tense, less emotional, less focused, and overall, more negative, despite being easier to referee and the players behaving more positively. Qualitative analyses of the video-taped interview footage indicated (i) substantial inter-individual variability regarding the extent of the effect of the empty stadiums on the subjective experience of emotions, (ii) consequently, different strategies to regulate emotions and arousal from suboptimal to optimal levels, both before and during competition, and (iii) interactions between reported emotions, arousal, motivation, self-confidence, behavior and performance on the pitch. In addition, non-verbal expressions of emotion were captured using fully automated AI-software that coded facial movements during interviews. The results of this exploratory facial expression analysis revealed varying degrees of arousal and valence in relation to the content of the statements during the interviews, demonstrating the convergent validity of our findings. Our findings contribute to the growing literature on the effects of football games without fans during the COVID-19 pandemic and provide insights into the subjective experience of professional football referees. Concerning referees and players alike, emotions are investigated as potential processes related to home-field advantage and performance in professional football by means of a multi-methods approach. Further, the combination of qualitative and quantitative measures—as well as verbal and non-verbal communication channels—can deepen our understanding of the emotional influence of (missing) spectators on the subjective experience and the behavior of sports professionals is discussed.

## Introduction

Due to the COVID-19 pandemic, many European elite football (a.k.a. soccer) leagues played large parts of the 2019/2020 and/or 2020/2021 season(s) without (or with severely limited) attendance of fans (so-called “ghost games”). From a sport psychology perspective, this situation presents a unique opportunity to study the influence of spectators on the experience and behavior as well as the performance of professional athletes and other stakeholders, such as referees in the most popular sport in the world. Previous studies have already shown a significant influence of the presence of fans on the experience of social pressure and emotional arousal of the players and referees, and subsequently on individual and team performance (e.g., Leitner and Richlan, 2021b; Pettersson-Lidbom and Priks, 2010; Scoppa, 2021).

Up to now, in the wake of the COVID-19 pandemic, research primarily investigated changes in home advantage, decision-making by referees, and the consequences of a lack of support from the ranks for various sports professionals. In this context, Leitner et al. (2022) recently summarized the empirical findings to date by means of a systematic literature review. The authors conclude from their analysis of 26 independent studies conducted during the COVID-19 pandemic that ghost games indeed have a considerable influence on home advantage in professional football. In fact, there was not a single study that found an increased home advantage in ghost games: six studies conclude “no change in home advantage”, two studies conclude a “slightly reduced home advantage”, eight studies conclude a “reduced home advantage” and ten studies conclude a “strongly reduced home advantage” in ghost games.

The first main reason for this effect seems to be a change in referee behavior. Specifically, it appears that referees favor the home team less in ghost games than in matches with regular attendance (e.g., Bryson et al., 2021; Leitner and Richlan, 2021b; Reade et al., 2021). For example, Leitner and Richlan (2021b) showed that home teams (but not away teams) were booked significantly more often with yellow cards for committing fouls in ghost games. This effect was independent of the course of the games and cannot be attributed to the overall weaker performance of home teams. Thus, referees assessed the play of home teams more objectively, reflecting a reduction of unconscious favoritism for the home teams compared to games with regular attendance.

As noted by Hill and Van Yperen (2021), however, the change in referee behavior has to be interpreted with caution, since in-depth analyses of the correctness of the decision-making processes are largely missing. This calls for more detailed behavioral and psychological analyses of the influence of the ghost games on referees and players, including individual and team tactical dynamics and playing styles.

This effect is most likely related to the changed acoustic scenery during ghost games. Even before the COVID-19 pandemic, several studies illustrated both naturally—on a smaller scale due to stadium closures following riots—and artificially—based on experimental settings in laboratories—that the acoustic atmosphere and feedback from the audience during football matches influences referees’ decisions and thus potentially the outcome of games (Goumas, 2012; Nevill et al., 2002; Pettersson-Lidbom and Priks, 2010). The second main reason for a reduced home advantage in ghost games seems to be the lack of emotional and motivational support from the stands for the home team (e.g., McCarrick et al., 2013; Scoppa, 2021; Sors et al., 2020).

Not only the effect of home advantage, however, seems to be affected by ghost games, but also the emotional behavior and experience of the protagonists themselves on the pitch is altered by the lack of (acoustic) atmosphere in the stadiums. Specifically, one study showed that there were fewer emotional, verbal discussions between players, referees, and coaches during ghost games in contrast to matches with regular attendance (Leitner and Richlan, 2021a). To summarize, all these studies and perspectives on the phenomenon of ghost games in the aftermath of the COVID-19 pandemic suggest that the audience seems to have a significant impact on the protagonists and the game itself.

What all these previous studies on football during the COVID-19 pandemic have in common is that they primarily rely on quantified data, such as performance statistics and match facts. In contrast, the present multi-study article investigates the effects of ghost games directly on the subjective experience of one of the most affected protagonists of ghost games: the referees. This is the logical next step in understanding the effects of spectators on actions, emotions, and cognitions in football. More specifically, it is required to move towards investigating the psychological processes underlying the observed effects within the people who are directly affected by ghost games.

Most of the studies conducted to date indicate that emotions experienced by referees and players, as well as the resulting interaction, behavior and decision-making processes between these groups on the pitch, seem to have been altered by the absence of the audience (Leitner and Richlan, 2021a, b; Nevill et al., 2002; Schweizer et al., 2023). Either in the context of home advantage, referees’ decision-making in contentious match situations, or the observation of emotional behavior during match breaks. The present work is the first of its kind to deal extensively and categorically with the subjective actions, experiences, and interpretations of professional football referees in the wake of the COVID-19 pandemic.

The present multi-study article investigates the self-efficacy, motivation, subjective experience, and observations (e.g., how the players behaved on the pitch from the referees’ perspective) of professional football referees during ghost games (compared with regular games). Self-efficacy denotes the person’s conviction that they can perform a goal-directed behavior (Bandura, 1982). Changes in self-efficacy due to ghost games would therefore provide an important indicator for changes of in-game behavior. Self-efficacy has already been shown to be an important predictor of referees’ behavioral and psychological match outcomes (Guillén and Feltz, 2011; Labudek et al., 2019; Myers et al., 2012).

In addition, we focused on two aspects of motivation that have been found to be predictive of performance in a work context: intrinsic and prosocial motivation (Grant, 2008). While intrinsic motivation denotes working for the enjoyment of the activity, prosocial motivation denotes working to help others (e.g., co-workers or customers). Finally, we explored arousal and excitement as candidate processes (Allport, 1924; Zajonc, 1965). Taken together, these processes have been at the center of the public and academic discussion surrounding ghost games in professional football (DerStandard, 2020a, b; Guardian Football, 2020; Hamilton, 2021; Leitner et al., 2022; UEFA.tv, 2020; Webb, 2020; ZDFsport, 2020).

To this end, we surveyed 36 professional football referees (one female and 35 males) from the Austrian Football Association (ÖFB) about their personal perception of the ghost games during the COVID-19 pandemic using an online questionnaire. Additionally, we conducted semi-structured, video-taped interviews with one referee and two players participating in the Austrian Bundesliga (i.e., the highest league in Austria) as a supplement to our collected survey data and to gain further and deeper understanding of the individual psychological processes underlying the survey results.

Based on our previous research on professional football during the COVID-19 pandemic, we postulated the following hypotheses for ghost games compared with games in front of a regular audience: (1) Overall subjective excitement and arousal is lower in ghost games, (2) ghost games are easier to officiate/play, (3) ghost games are subjectively less important/significant, (4) communication with referee colleagues/teammates is easier in ghost games, (5) motivation is lower in ghost games due to the lack of “atmosphere” in the stadium, (6) in ghost games, both referees and players are less emotional in critical situations, and (7) in ghost games, referees’ self-efficacy and self-confidence is higher and players’ self-confidence is lower.

Beyond this individual experience, we sought to explore a potential pathway how these individual experiences may influence in-game interactions between players and referees. We therefore explored the expected effects on arousal and emotions in facial expressions. Facial expressions are a common means to communicate affective states (Van Kleef, 2010); due to their visual nature, this communication channel is open during in-person as well as ghost games. In line with this view, there has been a recent surge in research on non-verbal behavior in sports (Furley, 2021; Furley et al., 2021; Furley and Memmert, 2021; Hopfensitz and Mantilla, 2019).

## Study 1: Referee survey

### Materials and methods

#### Participants

Thirty-six (semi-)professional football referees (one female, 35 males; 16 referees, 20 assistant referees) from the Austrian Football Association (ÖFB) participated in the study. The sample included referees with regular officiating appearances in the two highest football leagues in Austria (i.e., “First and Second Bundesliga”). Although many of the referees have regular daytime jobs, the leagues are largely considered “professional”—as indicated by a top ten ranking in the UEFA country coefficient and regular appearances of Austria’s top clubs in the UEFA Champions League.

The mean number of years of experience in these two professional football leagues was 8.0 years (SD = 5.2 years), with a mean average number of 24 games per season (SD = 5 games per season). The mean number of ghost games the participants were involved in was 23 games (SD = 5 games). To preserve anonymity, we asked for the age range instead of the exact age. Eight participants were in the age range between 25 and 29 years; 12 participants in the age range between 30 and 34 years; 12 participants in the age range between 35 and 39 years; and four participants were older than 40 years.

The call for voluntary participation in our study was sent out via e-mail by the official referee administration of the ÖFB to all semi-professional football referees in Austria (*N* = 64; participation rate = 56.3% of the population). The time window for participation was March 18 to April 7, 2021. The study was conducted in accordance with the Declaration of Helsinki and the general data protection regulation and was approved by the ethical review committee of the Paris-Lodron-University Salzburg.

#### Materials

The call for voluntary participation was sent out via e-mail and included a short letter and the weblink to our online survey hosted in the survey software Limesurvey (Limesurvey GmbH, Hamburg, Germany). Participants were instructed to respond honestly and spontaneously. The mean total processing time was 489 s (SD = 275 s). The survey consisted of an instruction including informed consent and a self-report questionnaire including 42 questions with the following structure:

*Part A*: Regular games (with supporters). Eight questions assessed self-efficacy using the German adaptation of the Referee Self-Efficacy Scale (REFS-D; Labudek et al., 2019; English version see Myers et al., 2012) and six questions assessed motivation to serve as a referee (three on intrinsic motivation, three on prosocial motivation; adapted from Grant, 2008).

*Part B*: Ghost games (without supporters). The questions from Part A were adapted to target ghost games instead of regular games, again assessing self-efficacy and motivation. The order of Part A and Part B was counterbalanced across participants. All answers to the questions from Parts A and B were given on seven-point Likert scales (1: not at all/strong disagreement—7: very much/strong agreement).

*Part C*: Direct comparison between regular games and ghost games. Eight questions assessed subjective experience and observations. These questions included subjective evaluation of experience, arousal, emotionality, focus, confidence, difficulty, and behavior of players and coaching staff. All answers to the questions from Part C were given on seven-point Likert scales as well, with 1 and 7 indicating the extreme values of the direction judgment (e.g., 1: less emotional—7: more emotional). The complete survey is presented in the supplementary material A.

*Part D*: One open commentary section and five general questions assessed participants’ demographics and experience as referees (age range, number of years of experience in the two highest football leagues in Austria, average number of games per season in these two professional leagues, number of ghost games involved as referee, assistant referee or fourth official).

#### Statistical analysis

For statistical analysis, Statistical Package for Social Sciences (IBM SPSS, version 26.0 for Windows) was used. To account for potential violation of the normality assumption, all comparisons were conducted using nonparametric paired-samples Wilcoxon signed-rank tests for Parts A and B (separate questions for regular games and ghost games, respectively) and one-sample Wilcoxon signed-rank tests for Part C (direct comparisons between regular games and ghost games within single questions). The effect sizes (Pearson *r*) for the Wilcoxon tests were calculated using the following formula: $r = \left| {\frac{z}{{\surd n}}} \right|$. Finally, we used the SPSS Macro MEMORE (Montoya, 2019) for exploratory within-participant moderation analyses. The threshold for statistical significance was set to *p* < 0.05.

### Results

#### Self-efficacy

**Fig. 1:**
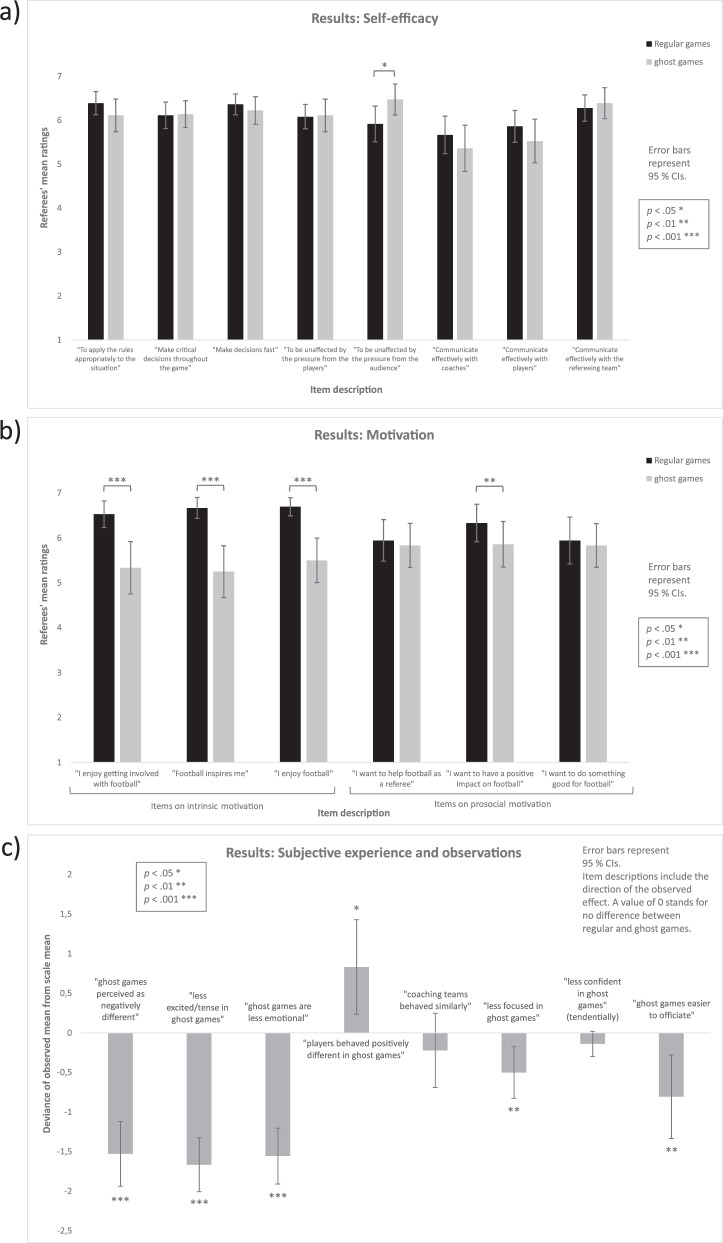
Results of the referee survey (means and 95% confidence intervals). **a** Self-efficacy in regular vs. ghost games. **b** Motivation (intrinsic and prosocial) in regular vs. ghost games. **c** Direct comparison of subjective experience and observations between regular and ghost games.

As shown in Fig. 1a, out of the eight items of the REFS-D, only one item (“To be unaffected by the pressure from the audience”) showed a statistically significant difference between regular games and ghost games, with ghost games eliciting higher self-efficacy, *z* = 2.30, *p* = 0.021, *r* = 0.38. One further item (“To apply the rules appropriately to the situation”) showed a descriptive trend with ghost games eliciting lower self-efficacy, but this difference was not significant, *z* = −1.78, *p* = 0.076, *r* = 0.30.

#### Motivation

As shown in Fig. 1b, all three items on intrinsic motivation showed a statistically highly significant difference between regular games and ghost games, with ghost games eliciting less intrinsic motivation. The three items were (i) “I enjoy getting involved with football”, *z* = −3.66, *p* < 0.001, *r* = 0.61, (ii) “Football inspires me”, *z* = −3.95, *p* < 0.001, *r* = 0.66 and (iii) “I enjoy football”, *z* = −3.82, *p* < 0.001, *r* = 0.64. Out of the three items on prosocial motivation, only one item (“I want to have a positive impact on football”) showed a statistically significant difference between regular games and ghost games, with ghost games again eliciting less prosocial motivation, *z* = −2.85, *p* = 0.004, *r* = 0.48.

#### Subjective experience and observations

Six items on subjective experience and observations showed a statistically significant difference with the experience in ghost games being (i) more negative, *z* = −4.51, *p* < 0.001, *r* = 0.75, (ii) less excited/tense, *z* = −4.85, *p* < 0.001, *r* = 0.81, (iii) less emotional, *z* = −4.77, *p* < 0.001, *r* = 0.79, (iv) less focused, *z* = −2.75, *p* = 0.006, *r* = 0.46, (v) easier to referee, *z* = −2.64, *p* = 0.008, *r* = 0.44, and (vi) players behaving more positively, *z* = −2.27, *p* = 0.023, *r* = 0.38. One item (“How confident were you?”) showed a descriptive trend with ghost games eliciting lower confidence, but this difference was not significant, *z* = −1.67, *p* = 0.096, *r* = 0.28. The remaining item (“Did the coaching teams behave differently?”) did not differ between ghost games and in-person games, *z* = −1.04, *p* = 0.297, *r* = 0.17.

For a visualization of the results, see Fig. 1c. The *y*-axis depicts the deviance of the observed mean for each of the eight items from the (theoretical) scale mean. A deviance of 0 signals no difference between regular and ghost games. For higher understandability, the item descriptions are included in the figure and were formulated according to the direction of the observed effect (e.g., “ghost games are less emotional”).

#### Open comments

The open commentary section of the survey provided the possibility for participating referees to give nuanced insights into underlying psychological processes and to emphasize aspects, which, from their personal perspective, are of particular importance or relevance. The answers to this section largely confirmed the quantitative results from the questionnaire and were as follows (original statements translated to English):

“It’s more fun with an audience! Still, you give 100% because it’s about football and making the right decisions”.

“I personally crave spectators again. I think everyone likes to be the center of attention a little bit. Furthermore, it promotes motivation”.

“It’s more difficult to motivate yourself. You also have to change the way of communication because everyone can hear everything”.

“The atmosphere in the stadium is missing. As this also pushes the referee!”

“Ghost games sometimes have a more pleasant effect, as maybe less pressure from outside is perceived, but this can also have negative effects, as the concentration or joy, motivation or tension falls off more quickly or leads to a certain routine or training match atmosphere. On the other hand, it can be perceived more positively, as the focus is forced on the game itself, unpopular decisions against the home team are probably made more unconsciously and without bias, and there is less distraction from the outside. It seems everyone is now more mindful of word choice and outward appearances, as these are now more prominent overall”.

“At the beginning it was very unfamiliar, and it was important to keep your concentration high and not lose it. Especially in games where there was little happening on the pitch as well. However, as the year went on, I got used to the situation and learned how to deal with it”.

“In the 2nd division, before the imposed ghost games, some matches with the second teams felt very similar because the stadium there was already similarly sparsely filled”.

#### Process analyses

To gain some insights into the processes underlying the unfavorable effects of ghost games on referee motivation, we conducted within-participant moderation analyses using the SPSS macro MEMORE (Montoya, 2019) and using an additive moderation model. We entered each motivation variable that showed significant effects as dependent measures in turn and tested the subjective experiences as potential moderators.

In order to avoid overfitting, we clustered similar variables and conducted separate moderation analyses for each cluster: (a) general evaluation: overall experience, players behaving more positively, overall evaluation; (b) own response: less excited/tense, less emotional, less focused. In order to reduce the number of necessary tests, we calculated the mean of all three intrinsic motivation items (*ɑ* = 0.92) since they all showed significant effects in the same direction. The effect of ghost games on the REFS-D item “To be unaffected by the pressure from the audience” was neither moderated by the general evaluation, all *p*s > 0.27, nor the subjective experience, all *p*s > 0.20.

The effect of ghost games on intrinsic motivation was moderated by being more focused during ghost games, *B* = −1.61, SE = 0.71, *p* = 0.03, and a relatively more positive overall experience of ghost games, *B* = 0.24, SE = 0.09, *p* = 0.01. Those referees who reported being relatively more focused during and having a more positive experience of ghost games reported smaller declines in intrinsic motivation due to ghost games. None of the other potential moderators were significant, all *p*s > 0.21.

Finally, the effect of ghost games on the prosocial motivation item “I want to have a positive impact on football” was moderated by a relatively more positive overall evaluation of ghost games, *p* = 0.009. Those referees who reported a more positive evaluation of ghost games reported smaller declines in prosocial motivation due to ghost games. None of the other potential moderators were significant, all *p*s > 0.110.

## Study 2: Video interviews

### Materials and methods

#### Participants

Concerning the additional semi-structured, video-taped interviews, the sample consisted of one referee and two professional players (one of them considered a “senior” player, the other in the middle of his career), all engaged in the Austrian Bundesliga (i.e., the highest football league in Austria). All had been refereeing/playing in professional football for at least 4 years. The participants gave informed consent. The main purpose of the video interviews was to supplement the survey data and to gain further and deeper understanding of the individual psychological processes underlying the survey results. The personal interviews provided the opportunity for the participants to freely provide their associations regarding the psychological factors around the ghost games.

#### Materials

The interviews were conducted and recorded via video-conference software (Zoom, 2013). They included the two authors FR and MCL and the respective interviewee. FR and MCL led through the interviews via semi-structured questions, which had been prepared a priori and were the same for all three interviewees. The interview questions were based on the survey questions but also provided additional opportunities for the participants to freely talk about their observations and feelings regarding the ghost games. These questions are provided in the supplementary material B. The interviews were conducted between May and August 2021, that is, after 12 to 15 months of playing without spectators.

Each interview began with a short introduction and information about the study. The main part of the interview consisted of questions regarding the subjective experience of the ghost games and the differences to regular games. The thematic sections of the interviews included (1) arousal, (2) difficulty, (3) importance/significance, (4) communication, (5) motivation, (6) noise/atmosphere, (7) player behavior, (8) referee behavior, (9) own behavior, and (10) self-confidence. Each interview ended with a short farewell.

This structure was used to guide the interviews, but participants were unrestricted in the depth and breadth of their answers. On some occasions, the interviewees shared important and crucial information without being prompted. In sum, the principal investigators made sure that the participants felt comfortable, and that the atmosphere of the interviews resembled a private conversation rather than a press conference or an interrogation. This was a difficult but decisive aspect, especially in the context of the work with football professionals, who are trained to be very careful with public statements and not used to talking openly and honestly about internal states. The interviews lasted between 23 and 55 min and had a total duration of 125 min.

#### Facial expression analysis

In addition to qualitative analysis, we used the AI software package FaceReader 9 (Noldus, 2021) to analyze participants’ facial expressions with regard to each topic in the interview. To this end, we cropped the videos so that only sections in which the participant was speaking on the respective topic were included, leaving out recordings when the questions were asked. Since interviews were semi-structured and participants sometimes provided answers to topics without being prompted, the exact question wording differed between participants. Accordingly, we combined all answers on one topic into one video per participant to analyze (analyses by question asked are available in the supplementary material).

The FaceReader software analyses each frame of the provided video input in the following way. First, the software uses a deep learning-based algorithm to identify a face in the image. If a face is found, the software then uses deep neural networks to model the face according to 500 key points (facial landmarks). This yields a vector model that describes the state of the face, which can then be categorized according to known emotion expression. The output includes so-called six basic emotions anger, disgust, happiness, sadness, fear, and surprise (Ekman and Oster, 1979) as well as more general indicators of valence and arousal. Due to the limited number of participants, however, we did not conduct statistical analyses, but rather report the results in a descriptive way.

Using software for emotion expression analyses has a number of advantages over traditional means of assessment (Kulke et al., 2020). First, software analyses are performed using standard video recordings, thus allowing for a maximally natural setting. For instance, our participants were partaking in a Zoom call, a common day-to-day activity during the COVID-19 pandemic. The study setting and analyses therefore have a high ecological validity, especially when compared with EMG measures that require putting electrodes on peoples’ faces. Traditionally, facial expressions have also been assessed using manual FACS coding (Ekman et al., 2002). Although coders may attain a very high accuracy, this requires extensive training and coding is highly time-intense. In contrast, software coding allows coding our videos on a frame-by-frame basis in an economical way.

The software advantages in terms of ecological validity and economy are paralleled by analysis accuracy. Validation studies of FaceReader and similar AI-emotion recognition software show that these analyses identify facial expression with similar accuracy as EMG measures (Kulke et al., 2020) and only slightly below traditional manual FACS coding (Dupré et al., 2020; Skiendziel et al., 2019; Stöckli et al., 2018). Directly speaking to the context of professional football, one study observed that software coding of players’ emotion expressions (anger and happiness) on official photos were good predictors of their team performance (Hopfensitz and Mantilla, 2019). In sum, FaceReader coding is a valid tool to analyze non-verbal emotion expressions.

### Results

#### Qualitative analysis of interview content

Qualitative analysis of the video-taped interview footage indicated (i) substantial inter-individual variability regarding the extent of the effect of the empty stadiums on the subjective experience of emotions, (ii) consequently, different strategies to regulate emotions and arousal from suboptimal to optimal levels, both before and during competition, and (iii) interactions between reported emotions, arousal, motivation, self-confidence, behavior and performance on the pitch. Below, we provide an individual analysis of the content of the interviews. See supplementary material for a complete transcript of the conducted interviews translated to English.

##### Player 1 (mid-career)

*Arousal, significance, and atmosphere*. The player describes the feelings before and during the first training sessions and ghost games after the COVID-19 lockdown as “very strange”, with a mixture of great joy and worry. For him, it was a novel situation (and feeling), which had an impact on his arousal before games. He was used to feeling the tension and atmosphere in the stadium on game-day before regular games (with supporters), and this was lacking in the ghost games. He mentions that this change was already noticeable during the journey to the stadium.

[…] it was certainly different to put yourself in that [tension] than now, when there was already a certain… yes, because of the presence of the other people… this alone creates a certain kind of tension. And um… That was the “before”, if I compare it roughly: the way to the game, the environment, the atmosphere, if you are in the stadium. That was definitely just very unfamiliar at the beginning, and it all seemed the same.

The player described that he had to develop a new pre-game routine to get himself into the optimal zone of arousal. He said that he used to be too relaxed and that he had to amp himself up before the ghost games. After kick-off, he used his first actions and tackles to get into his preferred state of tension. During the ghost games, he emphasizes internal and external “calmness” and that the lack of emotions from the outside (i.e., the spectators) resulted in relative absence of emotions on the pitch. There was nothing “electrifying”.

*Communication and atmosphere*. The team, including bench players and staff, tried to compensate for this lack of atmosphere and noise, but still, he reported the feeling that everything was a bit calmer and felt slower. This was especially evident in the time between two actions, where everything was more relaxed, and communication was easier. In regular games, he observed “waves of emotions” in the stadium, which were absent in the ghost games. In contrast, the atmosphere, mood, and energy levels were relatively constant during the ghost games and decreased even more towards the end of the games. In regular games, often the opposite is true.

To overcome this and to keep alertness, arousal, and focus high, the team worked with short-term performance goals for the last 15 min of the ghost games by means of tables. The player recalls situations playing abroad where they led 0–2 or 0–3 and the opponent scored a goal, which—in regular games with spectators—could be a potentially difficult and match-changing situation, due to the momentum and the atmosphere in the (away) stadium. In the ghost games, however, the team had no problem staying calm and composed and finishing the game without further goals scored by the opponent.

*Difficulty and own behavior*. Regarding his own behavior, the player noted that the missing reaction from the crowd after his good or bad actions may have influenced him subconsciously. To him, it felt easier to stop dwelling about his actions in the calmness of the stadium in the ghost games. In regular games, even if not focusing consciously on the crowd’s reaction, he is still aware of the noise from the stands, which, as he believes, can inhibit or spur him. In the ghost games, it was also easier for him to regulate and block out his own emotions if they were not amplified by the crowd’s emotions.

*Player behavior*. Concerning other players’ behavior, he noted that there were some who profited from the situation of the ghost games. Those players specifically showed less frantic behavior and better—not rushed—decision-making, especially in ball possession. He particularly mentioned attacking players who, in regular games, are full of adrenaline due to the crowd’s noise. With respect to defending players, the empty stadiums led to better communication among players and staff, and better coordination and faster orientation.

[…] They have definitely shown less hectic behavior. We’ve often had players who—let’s say—made a decision too hastily at certain moments, because the crowd has pushed them very hard because they’ve won a ball. Especially with our attacking players, offensive players, when we win the ball and they often have a lot of space, and they sprint [forward] and all of a sudden it gets loud in the stadium. And I don’t know… You’re full of adrenaline, so you make rash decisions and don’t play the ball. I rather had the feeling that often in such scenes some players could have made better decisions, because it seemed as if the pressure was less intense [in ghost games].

*Communication and referee behavior*. In addition, the player reported that the ghost games had an impact on the interactions with and the behavior of the referees. There were less provocations and less theatrics by players trying to influence referees with the help of the crowd’s social pressure. In sum, the ghost games were less emotional, with hardly any individual and mass confrontations, fewer provocations, and fewer attempts to influence the referee’s decisions in critical moments of the games. Ghost games were much calmer than regular games in this regard.

Especially as a player, you think that in an important, intense game, even small things can make the difference. If, for example, he [an opposing player] already makes the second foul or maybe you can trigger something through some emotion in the moment when the foul happens and maybe the referee or someone gives the player a yellow card, then you know for example: okay now the game changes again. And maybe as a result the yellow-red card… and that’s why I think that depending on—you can also exaggerate […] And he [the referee] consciously wants to calm down the game a bit in order to have less stress. I believe that when everything around gets loud, and you realize as a referee: “Now there are several… so reactions… now do I have to give him yellow or should I wait?” I don’t think that’s so easy, and I do believe that you can have an influence on that.

The referees themselves seemed to be deliberately more communicative in the ghost games compared with the regular games. Communication was also easier due to less noise. The referees seemed to be more relaxed and less pressured from the crowd and did not award yellow cards that easily. The player particularly noticed fewer “unnecessary” yellow cards due to hectic or rash behavior. The ability to better communicate with the referees may have led to fairer, calmer, and more factual games.

*Motivation and self-confidence*. Despite all these positive aspects, when asked directly, the player stated that he prefers the tension and the pressure of the regular games with spectators. For him, it is better to feel too tense than too loose and he is convinced that pressure helps him to live up to his full potential. He likes the atmosphere, the euphoria, the energy that the fans bring to the stadium. During the ghost games, he had to change his mental preparation in order to still get to the same intensity without the fans. For him, however, it was also an opportunity to grow into a leadership role on the pitch.

The player also mentions younger colleagues who might have benefited from the less tense atmosphere in the ghost games. In particular, new players who are a bit intimidated and first have to establish themselves as part of the team, probably found it easier without the external pressure from the fans. It takes time to find out how you (as a young player) and others react in certain situations. Self-confidence gradually builds up in this phase and the ghost games allowed for this process in a relatively safe environment, without the relentless emotional intensity of a full stadium. According to the player, this increase in self-confidence could also be perceived by the fans in consequent regular games.

[…] But I can imagine that for some young players—maybe in such a phase, where you are new in it [the club]—it was certainly rather an advantage, because they have not received pressure from the outside and all around and have been allowed to play and now they are already longer playing—a year or half a year—where it is a completely different feeling when you play in front of people, because they already have [integrated] into the team.

##### Player 2 (senior)

*Arousal and atmosphere*. The player reports that his arousal before the ghost games was significantly reduced compared with the regular games with supporters. He mentions the sound/noise in the stadium (even before the game has started), which leads to a certain “tingling sensation”. The more fans there are in the stadium the higher the arousal. Before regular games, he uses the warm-up in the stadium with the fans to get into his optimal zone of arousal. Before the ghost games, in contrast, he had to compensate for this lack of external stimulation through his personal mental preparation including listening to music and visualizing.

[…] you just try to create the right state of mind. So that you’re present right from the start, you’re in the game right away, and you don’t need any time to get started. From that point of view, I would say that it was very intense for us. Because normally, when you go out onto the pitch to warm up, there are already people in the stadium. There’s already a bit of a tingle, a certain sound in the stadium. So it’s not completely quiet, but rather you have a certain noise level and the more people there are in the stadium, the more tingling you have inside.

*Difficulty and motivation*. Once the matches had started, he did not notice any differences with respect to arousal or motivation between ghost games and regular games. According to him, it was just more difficult before the ghost games to get into the right performance state, that is, he had to work “more intensely” to get where he wanted to be mentally. During the ghost games, there was the same degree of ambition and motivation as in any other match. He mentioned the important role of the head coach, who emphasized motivation, focus, communication, and inter-individual support before and during the matches.

I’d say that, in my feeling, I was equally motivated. Like how your approach is, or what you plan to do. Very clear. *** He [the manager] did a really good job. He kept the concentration levels really high, so that we were very focused or actually got ourselves in the right mindset more than the spectators did. Just with the communication in the dressing room, on the pitch, the players who were or are on the bench, that they are also more vocal in the game with more enthusiasm and also provide support from the outside. That’s what you’re missing in a game with spectators… you really don’t notice that from the outside… whether it’s one of your coaches or someone else shouting something.

*Communication*. Communication between players was also the most important change in the training sessions before the ghost games. Other training topics did not change. Communication on the pitch between players was more intense and easier, due to the absent noise from the fans. For example, it was possible to communicate between the left and right sides of the field, which under normal circumstances is much harder. He also mentions that communication was “fairer” for each player (i.e., every player had the same possibilities to communicate).

*Own, player, and referee behavior*. Regarding his own behavior, the player states that there was not a big change in his communication, because he in general considers himself a good communicator. He and his teammates had to be a bit more cautious in the ghost games, however, because the referees were able to hear everything. Some other players had to work on their emotion regulation to curb their aggression towards the referee. This worked quite well and the referees themselves also seemed to be more relaxed due to the missing crowd, and immediately calmed down aggressive players. The player cannot recall any situation resulting in a huge upset or a really bad confrontation during the ghost games.

*Significance and self-confidence*. Like the other interviewed player, and despite these positive aspects, he likes the games with fans much more. It is more fun to play in front of a crowd, and especially cheering situations felt a bit awkward without fans. He had, however, no difficulty in accepting the situation with the ghost games and could focus on preparing for and performing the games on his usual level. He thinks that the whole team enjoys playing in front of a crowd—the more fans the better—and that it helps them to perform at their highest level. Interestingly, he noted that switching from regular games to ghost games was more difficult than the reverse.

##### Referee

*Arousal*. The referee describes the time of the first ghost games after the lockdown as a very odd situation and refers to the more general, novel circumstances related to the pandemic affecting everyday life. Social distancing, hygiene measures, etc. were also part of the pre-game procedure and resulted in fewer personal contacts and reduced time spent at the stadium. For him personally, this led to a reduction in arousal, but did not affect his motivation and joy to officiate games at the highest level.

*Communication, own, and referee behavior*. In contrast to the two interviewed players, the referee did not change his pre-performance routines. What changed for him—and for other referees as well (as he points out)—was the communication on the pitch. This was also part of pre-game referee briefings. For example, in the ghost games, the players and staff members could hear the communication within the referee team (one head referee, two assistant referees, one fourth official). Consequently, the team had to adapt to this novel situation by having clear communication guidelines. In addition, the referee team increased their communication to push themselves and to signal alertness and reciprocal trust.

Even more severe—at least from the personal perspective of the referee—was the situation that in the ghost games, the referees themselves could hear the players’ incivilities towards them. His strategy, in general and specifically in the ghost games, is to not take it personally, because he understands the emotions of the players and the pressure they are exposed to. The issue for him was more that he had to justify to external observers after a game why he was not stricter in sanctioning verbal insults against himself or his team.

[…] you hear everything we say as referees. Or even—actually worse for me—what the… what the players all say to us and we actually always go over it like this, because we know anyway that this is normal and that this is also okay, because I can’t imagine that there is someone who has something against me as a person. But this thing, to find the right balance, when you then look at it on TV afterwards and think “dude you really hear everything they said to us” and then they [the media reporters] ask why we don’t deal harder with them. This justification you have to do then why you don’t book them all off.

*Atmosphere and own behavior*. The calmer and less noisy atmosphere of the ghost games accommodated the referee’s personality, because he does not like the big gestures and the theatrical showmanship that is necessary for a referee to communicate his decisions in a full stadium. During the ghost games, he could communicate in a calm way and use all nuances of language and tonality, which in turn, had a positive impact on the interaction with the players. Another positive aspect of the ghost games was that he did not have to interact with the crowd in an artificially exaggerated way by clearly signaling his decisions to them as part of the show-like character that professional football has. This means that he did not have to “sell” his decisions to the crowd.

*Player behavior*. Regarding the players’ behavior, the referee noted that they seemed to really enjoy playing again (even in the absence of fans) and that they were more careful and a bit reserved with respect to fouls and other confrontations. They tried pulling cheap fouls less often and did not lie around or scream as exaggerated as is often the case in regular games with supporters. In general, they communicated calmer and more respectfully and did not obviously try to influence the referee’s decisions.

*Difficulty and atmosphere*. The referee thinks that the changed acoustics in the stadium are the main factor responsible for the different behavior on the pitch of both players and referees. Regarding acoustics and crowd noise, he added that it was potentially easier for the assistant referees/linesmen to call offsides, as it was easier to literally hear the moment when the pass was played. The referee also noted that the silence in the stadium helped to call fouls, as it was easier to hear when players were hit in tackles.

*Difficulty, significance, and motivation*. When asked directly whether ghost games were easier or more difficult to officiate, the referee gave a nuanced answer. Big, important games were easier because he could focus 100% on the task at hand instead of being distracted by the crowd. Smaller games were more difficult because his tension started to drift, and the crowd would have helped him to stay alert and focused. In general, he likes to be pushed by the fans. This also adds to his motivation and the subjectively perceived meaningfulness of events.

So I think even though I certainly don’t want to admit it like that now, that it is like that in my head. It simply gets less meaningful then. So that reminds you more like when you just hold a Landesliga [fourth division] or some similar match then, where there are just fewer spectators or otherwise somehow. This… this spectator thing… that does something to you. It doesn’t matter if you’re going somewhere internationally and suddenly there’s a stadium that has a certain look and no people are coming.

*Motivation and self-confidence*. With respect to self-confidence, the ghost games were beneficial for the referee because he could fully focus on his decision-making, and this led to the feeling that he increased his skills and became better from game to game. Because even in regular games he was already relatively calm, he had no big problems in adapting to the more relaxed situation of the ghost games. For long-term motivation, however, he stressed the importance of fans in the stadium, because they add to the meaningfulness by producing this unique flair that is a big part of professional football.

#### Quantitative analysis of facial expressions

**Fig. 2:**
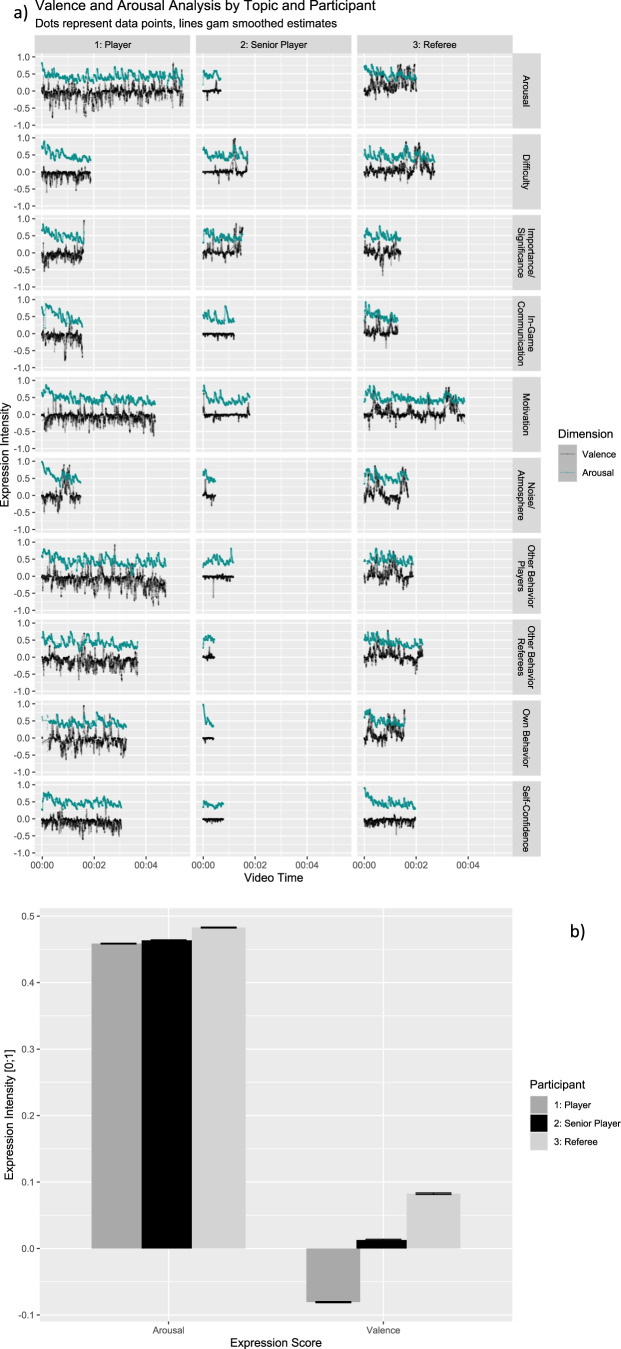
Valence and arousal measures derived from facial expression analyses using FaceReader 9. **a** Valence and arousal analysis by topic and participant. **b** Means of valence and arousal by participant. Error bars represent standard errors of the mean.

In addition to the qualitative analysis of the interviews, we captured non-verbal expressions of emotions using fully automated AI-software (Noldus FaceReader), that coded facial movements during the interviews. Based on the observed activation of 20 Action Units (AUs), FaceReader provides emotion expression intensity scores for each of the six basic emotions plus contempt, as well as summary scores of valence and arousal. Expression intensity scores and arousal vary from 0 (not present) to 1 (maximum intensity); valence scores range from −1 (maximum-negative intensity) to 1 (maximum positive intensity). As illustrated in Fig. 2a, this analysis revealed varying degrees of arousal and valence in relation to the content of the statements during the interviews, demonstrating the convergent validity of our results. For ease of interpretation, we provide a summary graph of the mean emotion expression per participant in Fig. 2b.

**Fig. 3:**
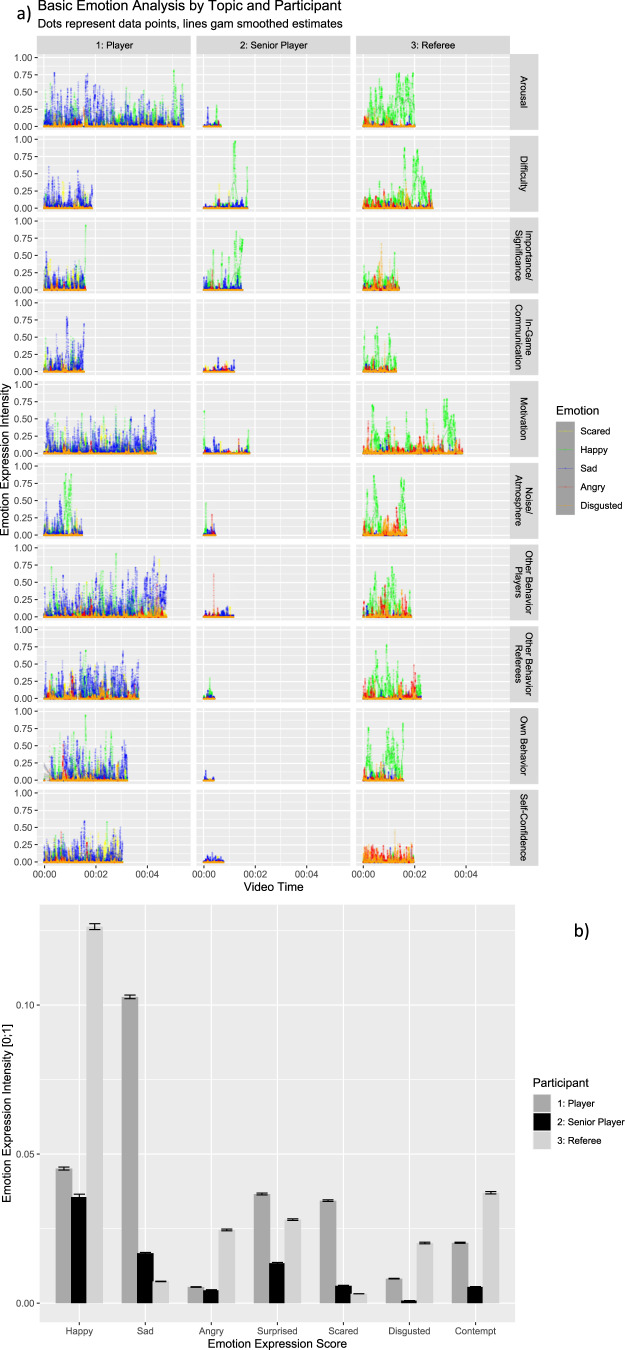
Basic emotion measures derived from facial expression analyses using FaceReader 9. **a** Basic emotion analysis by topic and participant. **b** Means of basic emotion expression scores by participant. Error bars represent standard errors of the mean.

Furthermore, we examined the relationship between each thematic section of the interviews (e.g., arousal, motivation, behavior) and the basic facial expressions of the interviewed players and referees according to Ekman’s basic emotions: happy, sad, angry, surprised, scared, and disgusted (Ekman et al., 2002). As shown in Fig. 3a, this analysis revealed significant inter-individual differences in facial expressions between participants (e.g., predominantly happy vs. predominantly sad, Fig. 3b), although the situations described and the interview setting were very constant.

Since the interviews were video-recorded, we could extract the speaking durations on the ten predefined topics (as described in *section* “*Video interviews*”*)* in our post-analyses. Table 1 depicts the speaking duration per topic separately for both players and the referee and the sum of speaking duration per topic across all interviewees. As shown in Table 1, Player 1 (mid-career) spoke the most about these in nine out of ten topics. In particular, talking about differences in arousal lasted 5:25 min with Player 1, whereas Player 2 (senior) talked about it a lot shorter (0:40 min).

**Table 1:** Speaking duration per topic in minutes and seconds for each interviewee (both player and the referee) and for the sum among all interview partners.

Speaking duration per topic (mm:ss)				
Topic	Player#1 (mid career)	Player#2 (senior)	Referee	Sum of speaking duration
Arousal	05:25	00:40	02:00	08:05
Difficulty	01:52	01:42	02:42	06:16
Importance/significance	01:37	01:31	01:23	04:31
Communication	01:33	01:22	01:17	04:12
Motivation	04:21	01:47	03:51	09:59
Noise/atmosphere	01:29	00:28	01:41	03:38
Other player behavior	04:46	01:09	01:52	07:47
Referee behavior	03:41	00:26	02:14	06:21
Own behavior	03:14	00:24	01:33	05:11
Self-confidence	03:03	00:46	01:57	05:46

Referring to the sum of speaking duration, motivation (09:59 min), arousal (08:05 min), and behavior of other players (07:47 min) are topics that were discussed the longest across all interviewees. Similarly, behavior of the referee (06:21 min), difficulty (06:16 min), self-confidence (05:46 min), and own behavior (05:11 min) were also topics of interest when talking about the effect of ghost games. Compared to this, the interviewees talked less about importance/significance (04:31 min), communication (04:12 min), and noise/atmosphere (03:38 min). Importantly, these latter topics—especially communication and noise/atmosphere—also came up when talking about the other topics.

The variance between the participants’ speaking time was accompanied by a great variance in their facial expressions (see Figs. 2a & 3a). While the referee was overall positive, as indicated by happy facial expressions and a higher valence score, Player 1 had substantially more negative emotional expressions, particularly sadness (Fig. 3b), and this was also reflected in overall negative valence score (Fig. 2b). In contrast, Player 2, a senior member of the league, showed relatively few emotion expressions, as indicated by a neutral valence score. There was some variation across topics (e.g., the referee talking about arousal vs. self-confidence) but this variation was substantially smaller than the variation between participants (see Figs. 2a & 3a).

In sum, these results indicate that theories about the impact of ghost games on football need to take into account the individual background of those involved. For referees, being able to focus on their tasks more may be a positive experience, especially when making close or contentious decisions. Among players, their tenure may be a key determinant of how they manage to cope with these changes. More broadly, the nuanced differences between participants highlight the importance of combining quantitative and qualitative approaches, especially when it comes to an in-depth analysis of emotions in sports.

## Discussion

Our findings contribute to the growing literature on the effects of playing without fans during the COVID-19 pandemic (e.g., Bryson et al., 2021; Leitner et al., 2022; Sánchez and Lavín, 2021; Wunderlich et al., 2021) and provide insights into emotions as potential processes related to home-field advantage and performance in professional football (Carron et al., 2005; Courneya and Carron, 1992; Jamieson, 2010; Nevill et al., 2002; Pollard, 2008). We discuss how combining qualitative and quantitative measures (Johnson and Onwuegbuzie, 2004; Johnson et al., 2007), as well as verbal and non-verbal communication channels (Furley, 2021; Furley and Memmert, 2021), can deepen our understanding of the emotional influence of (missing) spectators on the subjective experience and behavior of sports professionals.

Our initial hypotheses were largely confirmed, that is, (1) in ghost games subjective emotional excitement and arousal was lower, (2) ghost games were easier to play/officiate, (3) they were subjectively less important/significant, (4) communication with teammates/colleagues was easier, (5) motivation was (tendentially) lower due to the lack of “atmosphere” in the stadium, (6) both players and referees were less emotional in critical situations, and (7) players’ self-confidence was affected depending on maturity and referees’ self-confidence was higher.

The interpretation of the results from the qualitative and quantitative analysis of the interviews suggests that at least two central aspects (each with several sub-aspects) played a role in ghost games among the players and the referee, which will be discussed in detail in the following sections “Lack of external stimulation” and “Altered acoustic conditions”.

Our quantitative analyses of the underlying psychological processes indicated that cognitive variables were related to changes in referee motivation due to ghost games. In particular, measures of overall evaluation and experience were significant moderators of intrinsic as well as prosocial motivation effects. Being more focused during ghost games moreover contributed to smaller decreases in intrinsic motivation. In contrast, self-reported emotions or perceived changes of the players’ behavior had no consistent impact on these outcomes. One might therefore conclude that the influence of ghost games on referee motivation is mainly cognitive, with emotions playing a smaller role (Leitner and Richlan, 2021b; Pettersson-Lidbom and Priks, 2010; Scoppa, 2021; Sors et al., 2020).

Contrary to this conclusion, our qualitative investigation highlights the important role that emotions play. Specifically, our facial expression AI-based analyses (Kulke et al., 2020; Lewinski et al., 2014; Skiendziel et al., 2019) indicate that there is a substantial variation between individuals and subjects when it comes to expressing emotions regarding ghost games. Talking about the topic of motivation during ghost games, the interviewed referee exhibited positive emotion expressions (happiness), but also noticeable spikes in anger and disgust expressions.

Anger and disgust may be considered social emotions that commonly signal violations of cooperative or moral norms (Hutcherson and Gross, 2011), which our participants at least experienced temporarily when talking about their experience during ghost games. Apparently, spectators can influence these moral perceptions. This interpretation is in line with previous observations and speculations on the psychological effects of the ghost games on players and referees (Guardian Football, 2020; Hamilton, 2021; Leitner et al., 2022; UEFA.tv, 2020; Webb, 2020; ZDFsport, 2020).

More generally, the divergence of self-report and expression measures, as well as qualitative and quantitative findings highlight the importance of investigating ghost games using a multi-method approach (Johnson and Onwuegbuzie, 2004; Johnson et al., 2007). Regarding emotions, our findings may indicate that participants were unable or unwilling to provide accurate self-reports of their emotional experience during ghost games, leading to the null-relation to the observed motivation measures. In contrast, we did observe such emotions in the facial expressions of the interviewed referee. This may indicate that facial expressions are more accessible and/or less biased than self-reports (Kulke et al., 2020). We thereby provide a starting point to systematically investigate the role of facial expressions in future large-scale studies, thereby contributing to the growing literature on non-verbal behavior in sports (Furley, 2021; Furley and Memmert, 2021).

### Lack of external stimulation

The effects of the lack of external stimulation through absence of fans in the ghost games on subjective experience and behavior of players and referees can partially be explained within the framework of Social Facilitation Theory (Allport, 1924) and Drive Theory (Zajonc, 1965). According to this framework, the presence of other people (e.g., spectators in a football stadium) has a facilitating effect on the arousal and drive of the actors on the pitch. This increase in arousal and drive, in turn, influences performance, which—depending on the complexity of the task at hand—can be beneficial or detrimental. In ghost games, however, the external stimulation from the crowd is missing.

More modern accounts, such as the cusp-catastrophe model (Hardy, 1996), the individual zones of optimal functioning (IZOF) model (Hanin, 2000), or Kerr’s aggression theory (Kerr, 2004) emphasize a more nuanced relationship between emotional arousal and behavior/performance. The focus of these theories on individual ranges of emotions rather than a monotonous linear slope is in line with the substantial inter-individual variability of experienced emotions found in the present multi-study article.

Both the players and the referee emphasize that they adapted their actions before and during the ghost games to the changed situation of empty stadiums or that at least—according to their personal perception influenced how they dealt with certain situations. This is likely to have had an impact on individual and team performance and on match results. As previous research has found (Leitner and Richlan, 2021a, b) and Player 1 reported in this study, it is quite common to incite the home crowd to try to influence the referee in the player’s favor.

Similarly, the referee describes feeling more confident in his actions and more focused on his task because of the lack of external stimulation from the crowd (Guillén and Feltz, 2011; Myers et al., 2012). In addition, the referee reports that the reaction of the audience—which in turn can trigger a reaction in the players (and vice versa)—is anchored in his head. Thus, the referee talks about having to “sell” his decisions sometimes, for example, with non-verbal insistence to all participants to lend his latest decision more emphasis and to retain sovereignty over the game (Webb, 2020).

These aspects seem to play a subordinate role in the relatively empty stadiums in the ghost games, since “only” the players on the field and a handful of coaches and staff on the sidelines are present. Especially when it comes to close and ambiguous decisions and there is, for example, a high degree of uncertainty regarding the clarity of a foul or tackle, the immediate vocal reaction of the audience can have a crucial influence on the referee’s decision-making via unconscious social pressure (Cialdini and Goldstein, 2004; Nevill et al., 2013). This effect of crowd noise on referee decisions has also been shown experimentally (Nevill et al., 2002).

[…] there are 50/50 fouls where you say, “Yeah, never mind.” But if it’s already 60/40 [or] 70/30, then you should actually always whistle the fouls, because a foul is a foul. But if it’s really like that… Sometimes it makes it easier to accept these fouls… for me to accept them, because every audience, if everyone sees that, then the defender is the one to blame. So that can make some decisions easier, because you think to yourself: “Well, others see that, too, that you [the defender] were just clumsy.” Regardless of whether the other person wants that. So, it makes some decisions easier for me if I know that it just looks like that on the field.

Another aspect that both players and the referee agree on, is the subjectively reduced “importance” or “meaningfulness” of ghost games (DerStandard, 2020a, b). This could seem surprising in the highly professional world of elite football, but nicely illustrates the psychological processes at work. Similarly, Player 1 stated that the atmosphere during the ghost games had an impact on the subjectively perceived “importance” of the games, also because a certain monotony spread because the games all seemed very similar without the event character around football matches. In contrast, in regular games with supporters, home players were found to be feeling more motivated, whereas away players were found to be feeling more tense (Gómez-Ruano et al., 2021).

According to Player 1, this reduced importance may have had a positive impact on the development of young players, as they could benefit from being exposed to less pressure in ghost games than in regular games with spectators. This, in turn, could support integration to the team and familiarization with playing at the highest level, allowing players to gain confidence more quickly. Similarly, he had the feeling that—with less pressure from the stands—attacking players were not tempted to make hurried and rushed decisions but rather stay calm and find more thoughtful and ultimately better solutions.

The more experienced player, on the other hand, stressed that playing behind closed doors did not make much of a difference to him and, moreover, the coaching team had obviously responded well to the novel situation by putting an emphasis on team communication on the field, which is an important factor in team success (Eccles and Tenenbaum, 2004; Smith et al., 2013).

### Altered acoustic conditions

Similarly, both players and the referee describe that the changed acoustics during the ghost games play a considerable role in the execution of their actions, thinking, and feeling (Leitner and Richlan, 2021a, b; Nevill et al., 2002). Player 2, for example, reports altered pre-match emotions due to the silence in the stadium that occurs upon arrival well before the game. From a sports psychological point of view, existing routines and a possible adaptation of these play a decisive role here to achieve the usual mental performance level (Weinberg and Gould, 2019).

But not only before the match, also during the match the changed acoustics apparently played a considerable role. For both the players and the referee, silence means improved communication even over longer distances. In addition, the referee has better chances to interpret fouls and offside decisions (correctly). For example, hit sounds were much easier to hear in the case of failed tackles, or in the case of offside decisions, the assistant referee could better hear the sound when the ball was played and at the same time visually check whether the offensive player was offside or not. At the same time, the ghost games’ silence also means that players must control their emotions more strictly because any verbal lapse towards the opponent or the referee is easier to hear and could potentially result in a warning or booking.

Player 1 also emphasizes that there were marked differences compared to games with regular attendance due to the change in the overall acoustic situation. On the one hand, the players had to be careful not to say things that are normally easily overheard (intentionally overheard or actually overheard because of the noise level), and on the other hand, the loudness of their own voices could also be adjusted to the new situation.

[…] it was just like this that you have now heard everything. You didn’t have to be really loud now, you could have just talked normally and that’s also a little part of the emotions, when you get a little bit louder and want to say something to someone ***, because otherwise he can’t hear it exactly or things like that. That wasn’t so necessary now, I’d say, and that was certainly a point where I had to somehow [..] always make sure that I am always maintaining a certain tension […]

Particularly interesting against this background appear the further reports of Player 1. Due to the circumstance that in far advanced games, which were felt to be decided in their own favor, the inner tension threatened to drop due to the lack of external stimulation by the audience (Allport, 1924; Zajonc, 1965). The coaching team was aware of this situation and set countermeasures in the match preparations by emphasizing the importance of short-term process goals—a fundamental strategy in applied sport psychology (Weinberg and Gould, 2019) In a recent meta-analysis, process goals were shown to have larger positive effects on both performance and self-efficacy compared with performance goals and outcomes goals (Williamson et al., 2022).

[…] if you take normal games with spectators […] you have such phases in the game, in which it is briefly a bit calmer, depending on how you have played in the game and maybe you are playing it a bit calmer and more like keeping the ball or are we now consciously trying to score a goal… […] And then you’ve always noticed these mood swings in the stadium: “okay, now it’s a bit calmer, okay now is a bit more energy inside, now it’s a bit more energetic”. And you didn’t have that […] In the ghost games… […] it became even more relaxed, because maybe the game is already decided or you have already noticed from the opponent: “okay, there is absolutely nothing more coming, the game is over”. So [...] we worked with tables. For example, we wanted to stay as focused as possible in the last 15 minutes and did not want to concede a goal. We have set psychology goals for ourselves, so that we still maintain that [concentration] […]

## Conclusion

Research over the past two years on the absence of football audience points towards changes in both players’ and referees’ emotions, interactions, behaviors, and decision-making processes (Leitner and Richlan, 2021b). These alterations seem to have affected the home advantage, possibly through referees’ altered decision-making in ambiguous match situations (Leitner et al., 2022). Additionally—when the match was interrupted—less emotional behavior occurred on the pitch (Leitner and Richlan, 2021a).

In this mixed-methods article, we provide initial insights into self-reported subjective experiences (i.e., self-efficacy, motivation, and general personal observations and perceptions), actions, and interpretations of professional football referees during the COVID-19 related restrictions in professional association football. We also conducted in-depth semi-structured interviews with two professional football players and one referee (all three participating in the Austrian Bundesliga). The video-taped recordings were analyzed by means of a multi-methods approach using both qualitative and quantitative methods (Johnson and Onwuegbuzie, 2004; Johnson et al., 2007).

The unique insights gained by investigating both verbal and non-verbal forms of communication (e.g., Furley, 2021), along with the integrated use of quantitative and qualitative measures of subjective experience, contribute to a more in-depth understanding of football spectators’ influence on emotions, motivation, experiences, and behaviors of the predominantly involved parties (i.e., referees and players). Thus, the present mixed-methods article constitutes a first attempt to move beyond the typical use of match and performance statistics of previous studies to fully understand the underlying psychological processes of the stakeholders involved. Football constitutes an integral part of many people’s lives, and therefore understanding football can also mean understanding life.

## Supplementary information

Supplemental material

## Data Availability

The datasets generated and analyzed during the study are available from the corresponding author upon reasonable request.
