# Supplementary material for: Subjective experience, self-efficacy, and motivation of professional football referees during the COVID-19 pandemic
Source: Humanit Soc Sci Commun. 2023 May 8;10(1):215. doi: 10.1057/s41599-023-01720-z (PMC10166025; doi:10.1057/s41599-023-01720-z)
Supplement: Supplementary file 1 — Supplemental material [file 41599_2023_1720_MOESM1_ESM.docx]

**Subjective experience, self-efficacy, and motivation of professional football referees during the COVID-19 pandemic**

**Fabio Richlan^1,2*^, J. Lukas Thürmer^2,3^, Jeremias Braid^1,2^, Patrick Kastner^1,2^, & Michael Christian Leitner^1,2,4^**

^1^Centre for Cognitive Neuroscience, Paris-Lodron-University Salzburg, Austria

^2^Department of Psychology, Paris-Lodron-University Salzburg, Austria

^3^Department of Psychology, Ludwig-Maximilians-University Munich, Germany

^4^Salzburg University of Applied Sciences, Salzburg, Austria

*** Correspondence:**

Fabio Richlan

Fabio.Richlan@plus.ac.at

**Supplementary material**

**PART A**

**Referee survey**

***GHOST GAMES***

Now please think about "**ghost games**" in which you have acted as referee / assistant / 4th official.

Please think about how **confident** you felt as a referee / assistant / 4th official in these "ghost games". Please answer the questions below **honestly**. There are no "right" or "wrong" answers. Only your subjective perception in the course of "**ghost games**" in which you were actively involved counts.

Regarding your *tasks* in the course of "**ghost games**," how **confident** were you about your ability to...

1. apply the rules appropriately to the situation.
   (not at all) 1 – 2 – 3 – 4 (neutral) – 5 – 6 – 7 (very much)
2. make critical decisions throughout the game.
   (not at all) 1 – 2 – 3 – 4 (neutral) – 5 – 6 – 7 (very much)
3. make decisions fast.
   (not at all) 1 – 2 – 3 – 4 (neutral) – 5 – 6 – 7 (very much)
4. be unaffected by the pressure from the players.
   (not at all) 1 – 2 – 3 – 4 (neutral) – 5 – 6 – 7 (very much)
5. be unaffected by the pressure from the audience.
   (not at all) 1 – 2 – 3 – 4 (neutral) – 5 – 6 – 7 (very much)
6. communicate effectively with coaches.
   (not at all) 1 – 2 – 3 – 4 (neutral) – 5 – 6 – 7 (very much)
7. communicate effectively with players.
   (not at all) 1 – 2 – 3 – 4 (neutral) – 5 – 6 – 7 (very much)
8. communicate effectively with the refereeing team.
   (not at all) 1 – 2 – 3 – 4 (neutral) – 5 – 6 – 7 (very much)

Regarding your motivation in the wake of "**ghost games**," how strongly do the following statements apply to you:

1. I enjoy getting involved with football
   (strong disagreement) 1 – 2 – 3 – 4 (neutral) – 5 – 6 – 7 (strong agreement)
2. Football inspires me
   (strong disagreement) 1 – 2 – 3 – 4 (neutral) – 5 – 6 – 7 (strong agreement)
3. I enjoy football

(strong disagreement) 1 – 2 – 3 – 4 (neutral) – 5 – 6 – 7 (strong agreement)

1. I want to help football as a referee
   (strong disagreement) 1 – 2 – 3 – 4 (neutral) – 5 – 6 – 7 (strong agreement)
2. I want to have a positive impact on football
   (strong disagreement) 1 – 2 – 3 – 4 (neutral) – 5 – 6 – 7 (strong agreement)
3. I want to do something good for football

(strong disagreement) 1 – 2 – 3 – 4 (neutral) – 5 – 6 – 7 (strong agreement)

***REGULAR GAMES***

Now please think about "**regular games**" (with an audience) in which you have acted as referee / assistant / 4th official.

Please think about how **confident** you felt as a referee / assistant / 4th official in these "regular games". Please answer the questions below **honestly**. There are no "right" or "wrong" answers. Only your subjective perception in the course of "**regular games**" (with an audience) in which you were actively involved counts.

Regarding your *tasks* in the course of "**regular games**," (with an audience) how **confident** were you about your ability to...

1. apply the rules appropriately to the situation.
   (not at all) 1 – 2 – 3 – 4 (neutral) – 5 – 6 – 7 (very much)
2. make critical decisions throughout the game.
   (not at all) 1 – 2 – 3 – 4 (neutral) – 5 – 6 – 7 (very much)
3. make decisions fast.
   (not at all) 1 – 2 – 3 – 4 (neutral) – 5 – 6 – 7 (very much)
4. be unaffected by the pressure from the players.
   (not at all) 1 – 2 – 3 – 4 (neutral) – 5 – 6 – 7 (very much)
5. be unaffected by the pressure from the audience.
   (not at all) 1 – 2 – 3 – 4 (neutral) – 5 – 6 – 7 (very much)
6. communicate effectively with coaches.
   (not at all) 1 – 2 – 3 – 4 (neutral) – 5 – 6 – 7 (very much)
7. communicate effectively with players.
   (not at all) 1 – 2 – 3 – 4 (neutral) – 5 – 6 – 7 (very much)
8. communicate effectively with the refereeing team.
   (not at all) 1 – 2 – 3 – 4 (neutral) – 5 – 6 – 7 (very much)

Regarding your *feelings* in the course of "**regular games**" (with an audience), how strongly do the following statements apply to you:

1. I enjoy getting involved with football
   (strong disagreement) 1 – 2 – 3 – 4 (neutral) – 5 – 6 – 7 (strong agreement)
2. Football inspires me
   (strong disagreement) 1 – 2 – 3 – 4 (neutral) – 5 – 6 – 7 (strong agreement)
3. I enjoy football

(strong disagreement) 1 – 2 – 3 – 4 (neutral) – 5 – 6 – 7 (strong agreement)

1. I want to help football as a referee
   (strong disagreement) 1 – 2 – 3 – 4 (neutral) – 5 – 6 – 7 (strong agreement)
2. I want to have a positive impact on football
   (strong disagreement) 1 – 2 – 3 – 4 (neutral) – 5 – 6 – 7 (strong agreement)
3. I want to do something good for football

(strong disagreement) 1 – 2 – 3 – 4 (neutral) – 5 – 6 – 7 (strong agreement)

***DIRECT COMPARISON***

If you compare "**ghost games**" and "**regular games**" (with an audience) in your role as referee / assistant / 4th official - in general. How do you evaluate the following questions? Please answer the questions **honestly**. There are no "right" or "wrong" answers, only your subjective perception counts.

1. How did you perceive "ghost games" compared to "regular games" (with an audience)?

(negatively different) 1 – 2 – 3 – 4 (similar) – 5 – 6 – 7 (positively different)

1. How excited / tense were you in "ghost games" compared to "regular games" (with an audience)?

(less excited/tense) 1 – 2 – 3 – 4 (similar) – 5 – 6 – 7 (more excited/tense)

1. How emotional were you in "ghost games" compared to "regular games" (with an audience)?

(less emotional) 1 – 2 – 3 – 4 (similar) – 5 – 6 – 7 (more emotional)

1. Did the players behave differently in "ghost games" than in "regular games" (with an audience)?

(negatively different) 1 – 2 – 3 – 4 (similar) – 5 – 6 – 7 (positively different)

1. Did the coaching teams behave differently in "ghost games" than in "regular games" (with an audience)?

(negatively different) 1 – 2 – 3 – 4 (similar) – 5 – 6 – 7 (positively different)

1. How focused were you in "ghost games" compared to "regular games" (with an audience)?

(less focused) 1 – 2 – 3 – 4 (similar) – 5 – 6 – 7 (more focused)

1. How confident were you in "ghost games" compared to "regular games" (with an audience)?

(less confident) 1 – 2 – 3 – 4 (similar) – 5 – 6 – 7 (more confident)

1. To summarize: How was it as a referee / assistant / 4th official to officiate "ghost games" - compared to "regular games" (with an audience)? It was ...

(easier) 1 – 2 – 3 – 4 (similar) – 5 – 6 – 7 (harder)

***CLOSING QUESTIONS***

I would like to make the following comments on the subject of "ghost games":

*Open*

Age:

*Category*

*20 - 24 / 25 - 29 / 30 - 34 / 35 - 39 / 40 - 45+*

Referee / Assistant / 4th official since (in years)

*Number input*

Average assignments as referee / assistant / 4th official per year

*Number input*

**PART B**

# Player 1 (mid-career) transcript

IV1 = Interviewer 1, IV2 = Interviewer 2, *PL1 = Player 1*

*** = excluded parts due to possible inferences

---GREETINGS & EXPLANATIONS—

IV1: In general, how did you experience these ghost games, if you just compare them and think about it: How did you experience that back then? It is just a general question: What was it like for you when there were suddenly ghost games?

*PL1: Um, basically it was a very strange feeling. Especially also when... it has already started after the Corona break... just as the group training has started. It was a mixture of great joy, because we saw other people again, but also with the restrictions, with all the rules that were… it was just always in your mind "okay, you have to pay attention to every little thing, that nothing happens, that you don't infect yourself, that someone catches it [the virus] or maybe the restrictions are tightened again.” The phase at the beginning was really strange and completely new. So, we couldn't imagine anything beforehand and how it would be... we train with training groups of six. You're not allowed to see other groups - that kind of thing - and on the one hand… that was a mixture: something nice, because you still saw at least five of your teammates, as you were assigned in [groups] and you had a little bit of your soccer life again with the training, even if it was completely different. We were not allowed to touch each other in the group of six. And on the other hand, it was just like this: “Okay, how long is this going to last? How long will it last and when will a bit of normality at least come into the training?” Because we knew that the thing with the spectators and everything else would take a long time. And this was the beginning, where I would have said, where this strange feeling has already come up a bit. And in direct comparison on the championship groups in comparison, where just simply... you arrive at the game and there is already a bit of tension before. Simply through the journey, then... you arrive at the stadium, and you realize: “ok there's atmosphere, something is already building up, people are there because they want to see us play, because they want to see a cool game, because they want to be entertained.” And we have, of course, also through this atmosphere that arises... on the way there... you get in a certain tension and that was certainly a big difference, if you compare… if you go to the game now, it just seemed, at the beginning especially, as if it were now a game in the preparation phase [before the start of the championship] and you are now playing somewhere and there are maybe - I don’t know - 100 spectators and there were also maybe 100 people in the stadium in total with all the media and so on. And this atmosphere there, this tension already before the game, already on the way there, has definitely changed and I think... so I'm now a player who did not find it difficult, but that there are certainly players who go into the game with a certain tension… it was now certainly not so easy, because you have had to bring yourself in, develop a new routine, a new, let me say, with what you bring yourself into the “mode” a little bit beforehand, like: “okay now is maybe an important game, because every game is the same, no matter what would have been now.” Whether we take the very first test match *** or then the very first *** There was no difference. Of course, it was in our minds: *** Of course, everyone wanted that at all costs, but it was certainly different to put yourself in that [tension] than now, when there was already a certain... yes, because of the presence of the other people... this alone creates a certain kind of tension. And um... That was the “before”, if I compare it roughly: the way to the game, the environment, the atmosphere, if you are in the stadium. That was definitely just very unfamiliar at the beginning, and it all seemed the same.*

IV1: And if we now look at what happened during the game: so maybe - if you think about it - you were somehow in a different mood, maybe you were less excited, for example, because you knew that no one was there now or that no one was watching. Maybe there was a subjective difference. I don't mean that in a negative way, this "being excited”.

*PL1*: Mhm, I understand.

IV1: So, what you said before. Were you maybe less excited or differently excited? How was that for you? How did you experience that?

*PL1: I was definitely more on the relaxed side than on the tension side. Just as you have described now. Rather the positive [kind of] tension, not the nervous one. So that you think to yourself: “okay, now it starts, the referee blows the whistle, there is a bit of atmosphere and now the game begins.” For me, I rather had to look that I am not too loose in the game or that I nevertheless bring myself in a certain tension and for me it was mostly like... for me it was always the first actions, the first duels. Because in the game... for us it's really like that - because people have often asked me: “yes, in the game, for example, when there are 30,000 people there [in the stadium]. What do you notice there? Or what do you think in this situation?” And then I often said that after the game it's difficult for me to tell someone what I was thinking during that phase of the game, because it just goes by so quickly, you're focused on the individual actions, and afterwards it's like this: when there's nothing going on [in the stadium], every action somehow feels like it takes a little longer. Somehow, in the time, when for example one action changes into another - whether it's an out, a free kick or something like that - you have this calmness and this calmness has somehow triggered... uhm not this electrifying thing, when for example you really want to score a goal, an action happens and then the action is cheered or you win the ball or you have a shot on the goal and even if it's not on the goal, there's always some emotion from the outside and that was just different. So, it's true... this emotion didn't really arise either. Okay, for example, you cleared the ball and then you already... then you had your team, the coaching staff from outside, also those from the bench, where we also specifically tried to do something… but this was just a bit missing and therefore everything was already a bit, I don't want to say that the tempo was slower, but now from action to action was somehow... you just felt this space, this in-between-time a bit more, everything was a bit more relaxed. Plus, it was just like this that you could hear everything now. You didn't have to be really loud now, you could have just talked normally and that's also a little part of the emotions, when you get a little bit louder and want to say something to someone ***, because otherwise he can't hear it exactly or things like that. That wasn't so necessary now, I'd say, and that was certainly a point where I had to somehow - as I said before - always make sure that I am always maintaining a certain tension, because it didn't change in the game. That's the next thing, if you take normal games with spectators, for example, you have such phases in the game, in which it is briefly a bit calmer, depending on how you have played in the game and maybe you are playing it a bit calmer and more like keeping the ball or are we now consciously trying to score a goal... totally to score a goal or perhaps also consciously want to prevent a goal with all power. And then you've always noticed these mood swings in the stadium: “okay, now it's a bit calmer, okay now is a bit more energy inside, now it's a bit more energetic.” And you didn't have that either [in games without spectators], for example. In the ghost games... that was so... yes, almost one level and it was towards the end... it became even more relaxed, because maybe the game is already decided or you have already noticed from the opponent: ”okay, there is absolutely nothing more coming, the game is over.” So, it was more like this for us, what we tried to… we worked with tables. For example, we wanted to stay as focused as possible in the last 15 minutes and did not want to concede a goal. We have set psychological goals for ourselves, so that we still maintain that [concentration], because in this way you have noticed ... maybe you lead 2-0 or 3-0 and you maybe have [i.e. play against] an opponent, where you play away maybe. But that's the next thing, there's absolutely no difference and then maybe something would have come up if [the opponent] would have scored a goal or would have had a good action or a good phase and especially if they would have scored a goal in their good phase, then maybe it could have been a deciding factor. But we have had games where the opponent - I think there were one or two games - also scored a goal in a good phase, but, quite honestly, we were just relaxed. So, it was like: “ok stupid, we were unfocused. Surely annoying, we did not want that, but then...” so the opponent did not really come ahead, where it otherwise could have been a bit dicey. But it was like that we often have thought, especially at the end of the game, it is rather like: “okay you know, we play the game like this until the end.” There was rather a bit less of the tension again.*

IV1: Very interesting, very cool. Thank you very much for that. Now very specifically, if I may ask: If you think about yourself, did you somehow behave concretely different in the ghost games, if you compare it. With an audience, without an audience. So, does something concrete come to your mind? I know it's difficult now in retrospect to say that I noticed something. In retrospect, I did that differently, I behaved differently than I normally would have.

*PL1: Um, in the game… I think rather that... If one takes it in such a way that the fans perhaps always unconsciously play a role, then it is that someone has no reaction from other people to an action of you. I would rather think of something like that, that you no longer think too much about what you may have done wrong, or you may have done right. When there was just this calmness... then it was just like: “okay I just did something stupid and was pointed out by my teammates and coach.” But you don't perceive such a reaction from a crowd who says: “Phew, what is he doing?” Or something like that. Even if you think:”Okay, I don't care.” You still get the reaction and I believe that this nevertheless could inhibit you but, in this phase, you just have continued the next action. *** Okay I didn't get the right... subconsciously a right reaction now. But if I still don't get the ball across in a full house the fourth time, more and more people will say: phew what's he doing?I think that maybe this would be where you could say that as a player your short-term focus just changes on what you just did and on the action. Because, of course, you notice everything around you and you notice even if there are 10,000 - 20,000 spectators in the stadium, you also get some reactions. The same thing: when you have done something well, you get some emotion from outside, where they clap or shout. That's also something that pushes you or you think to yourself: “okay, great action, that was good, I'll make sure that I continue like this.” And in the ghost games you had no reaction, no matter whether you did something good or bad. There was just clapping from outside. You got a little feedback from your team, but that's not the same as 20,000 people cheering when you score a goal or play a good ball or win the ball. And I would have said that from the emotion of the individual actions or reactions - maybe from yourself - that something has changed here. That you no longer perceived it so consciously and let it get to you. But you simply thought: “Yes, go on.” You suppress the emotions faster than if the reaction to your actions would be larger or louder.*

IV1: Did you also notice that about your teammates? Did you notice that one or two of them behaved differently than they normally would? Just as you just described. Did you notice anything? You know the people quite well, how they behave on the field. Did you think at some point that he [a teammate] was behaving differently and what was different about that?

*PL1: Yes, for sure. I would have said that there are certainly players who benefited from that phase, who have shown a changed reaction in terms of reaction [from the stands]. Rather the reaction in the game was... they have definitely shown less hectic. We've often had players who - let's say - made a decision too hastily at certain moments, because the crowd has pushed them very hard because they've won a ball. Especially with our attacking players, offensive players, when we win the ball and they often have a lot of space, and they sprint [forward] and all of a sudden it gets loud in the stadium. And I don't know... You're full of adrenaline, so you make rash decisions and don't play the ball. I rather had the feeling that often in such scenes some players could have made better decisions, because it seemed as if the pressure was less [in ghost games]. At this point I really think more of offensive players than defensive players... I noticed a difference in defensive players, but it was a positive one: The communication was better because you could just point something out to someone much quicker and from outside from the bench, if anyone misses something, you could just yell to them to turn around and look. That was something positive that just worked faster to get your orientations faster. Now *** that was just extremely... yeah just an improvement there. And in the offense, I would have rather said that the... yes, when you win the ball, you feel euphoric and good and you get an energy boost, but then often - I think - because of what's going on all around, that it gets loud, a bit of a mood arises, you often don't stay in that mode and that you play the ball an emotion further. And I just noticed - now on the offensive [side] - that this phase of hecticness was a bit more controlled and the decisions were made better than before. Maybe that was only in games... but from the feeling, I would have said that.*

IV1: Because you mention this hectic pace and this control. Specifically, have you noticed that about fouls as well? Or would you say that nothing is different about that? For example, if there were wrong decisions or something like that?

*PL1: Well, yes, that's what I would have said too, that there is definitely a change. Above all, I would be interested in how the referees would have seen it. Because from my point of view as a player, I have the feeling that... with spectators you can always bring a certain kind of provocation into the game. Whenever there is a foul... So okay, there was a foul now. Then it's a game, which is maybe very combative and intense. If the atmosphere is like that and the people react to everything, then I think you can set a provocative stimulus to influence things and decisions a little bit. For me as a player, I noticed: “Okay, there was a foul.” And then maybe a player - one behaves like this, the other behaves like that - makes a show or exaggerates a bit. But then it was over quite quickly. There has briefly been the moment, but then... that something really comes up... I can't even remember a game that was like this. Of course, what you have had now was a certain provocation by the teams through the cheering and so on. Or if you score a goal and the whole bench... we shout and clap and the others sit there 10 meters away and hear everything, then it's a bit of a... it's annoying, it's provocative. Even if it wasn't in your direction, but indirectly it goes in your direction. *** That has provoked us, although it is like: “Okay we lost and it was a bad game.” But that was *** and it was something, when you concede a goal, which has really annoyed you. And when you're in the stadium now, the other side is cheering and the ball is in front again, then there's a bit of an atmosphere again, but then it's forgotten again. And the moment when it just happens, it was purely from the provocative side more intense than if it were the fans and the guest block that cheered. That was the case. The same with the fouls, to bring it back to those. I would have said that because of this [the ghost games] a conscious participation as a player to provoke or create something definitely has not happened as easily as with a big crowd [in the stadium]. That’s what I have noticed. Because simply… Okay it's a foul and he comes to you, then you shake hands and maybe say something. But that something provocative arises in a group, that a few people would run towards [you or the referee] because something is going on, because the player wants another confrontation, I do not think ... I do not know anything. No game where that would have happened. So that certainly plays an interesting role, when the broad masses bring in an emotion in such actions, that you often take this with you. That was definitely not the case. It was certainly much calmer in this regard if we now look at the topic of "fouls" and so on.*

IV2: May I dig in there for a moment. You said that this is definitely a conscious process, that sometimes you try to take the spectators with you, that maybe the referee is influenced?

*PL1: Well, definitely. Especially as a player, you think that in an important, intense game, even small things can make the difference. If, for example, he already makes the second foul or maybe you can trigger something through some emotion in the moment when the foul happens and maybe the referee or someone gives the player a yellow card, then you know for example: okay now the game changes again. And maybe as a result the yellow-red card... and that's why I think that depending on - you can also exaggerate, then you get the yellow [card] - but just when you realize: “okay now the referee is already maybe - let's say - not sure: should I give it [the yellow card] to him or not? Because he thinks: okay I could give it or I know what he thinks.” And maybe at this moment you give him a certain stimulus and say: "Hey, that can't be [fair]" or I don't know, "Didn't you see that" or " it’s this player again [who makes the foul]". I don't know what you're saying. And he consciously wants to calm down the game a bit in order to have less stress. I believe that when everything around gets loud, and you realize as a referee: “Now there are several… so reactions… now do I have to give him yellow or should I wait?” I don't think that's so easy, and I do believe that you can have an influence on that.*

IV1: And did you also observe whether the referees behaved differently in the ghost games?

*PL1: *** That, yes. Because otherwise in a game you… if everything is full and it's loud, I also believe that the referees were consciously a bit more communicative in the ghost games than in a full stadium. Because I think that the referee does not want to spend a long time with the situation and just makes the decision and wants to continue. And also, of course, from the outside. He will also be in a certain concentration mode and make sure that he doesn't miss anything and continues. I would have said that communication has certainly changed. And I know from the feeling, I think, they have now not given a yellow card that quickly - seen from my team… Now in our games there were not always such unnecessary yellow cards. I don't know how to describe it. It was more like that, I think, I could attribute to the whole mood, to the whole level of such a game, that also for the referees when the game is running out – in the end - it flattens a little bit. Because ok, the opponent doesn't really have anything, if he doesn't score a goal or something, that gives him additional motivation and maybe builds up a bit of tension again. I also think that especially towards the end of the game nothing happens anymore, and it was also no longer that intense for the referee, where he no longer had so many situations where he must have the full overview. I think that perhaps that has also changed. Because I can't remember any game that was so extreme in the end - if I take the league as an example… where it would have been extremely hectic. I would have rather said the communication has certainly changed. And perhaps also in connection with the general tension level of a game, especially towards the end, that this has also changed again. But it is still different from one referee to the other referee. One is generally a bit more communicative and the other stays on his line and says: “No, I don't want to talk now, and I don't have to deal with you, I don't have to give you any justification.” This is something that has not changed. So, once it is like this and then like that. But yes, basically many players will probably see it differently, *** most were generally rather communicative with one/two exceptions. From there I would have seen the communication as the biggest change, because the referee is louder, and you hear them better. You can briefly say something to him and maybe also in the follow-up action... maybe briefly discuss or talk or ask questions. I would have said that now in this field, which had been salient.*

IV1: In general, in summary: If you had to evaluate it now. Would you say that these ghost games... Did you find it easier or harder? When you think about it - honestly - do you prefer it with fans, or do you prefer it without fans? Or does it seem easier or harder to you? This is just a subjective feeling.

*PL1: Well, I'm one who needs this tension and this pressure. So, it's definitely better for me if I... I'd rather have too much tension than too little. Because I think I'm just that kind of guy... I think that could bring me to my limit, what is possible now on this day and would be possible to bring on the field. So, I think that I, personally, find in the long term that it is really bad without fans. It was already difficult. And a good example is - for example - the last game, where the fans were there. *** But the feeling, even though there were only 3,000 people, was quite different and that's exactly what I said before. There is simply a different atmosphere and even if there are only a few people. You come to the stadium and have a quick look at the place and just see people there. You realize: “Okay, you can already feel this euphoria and energy that people bring with them when they go to the stadium.” And from that point of view, I couldn't imagine it [ghost games] in general. I couldn't have imagined it back then either. I have always said ghost games… when you have seen them as a punishment, clubs have had to make ghost games. That was also just to watch on TV... has been the... I don't know... something [bad]. And as a comparison, I was... well, I had to change something in my mental preparation through the ghost games, in order to maintain this tension and also to build it up. And it is of course completely different for me, if I now know: “okay, we are playing in an interesting game and people are there and it's full, even if it's still a league game and there are 10,000 people there, it's still something.” It's still from [my] feeling something that I need. It's definitely something that will rather enhance my performance, for me personally, than inhibit it. For me. But I can imagine that for some young players - maybe in such a phase, where you are new in it [the club] - it was certainly rather an advantage, because they have not received pressure from the outside and all around and have been allowed to play and now they are already longer playing [i.e. for a longer time] - a year or half a year - where it is a completely different feeling when you play in front of people, because they already have [integrated] into the team... Because I know how it is when you come into the team as a young player and at some point, you automatically have the feeling: “ok, it's normal now that I'm there, my routine is normal, the way I act is normal.” And it's just like when you come, you just need a little bit of time, you're a little bit more reserved. That's when you look at how things work, look how other people react to me, how do I react to things. And I think that could be an advantage for some players who have joined during the Corona phase, who are already over this phase and can now appear with more self-confidence in the team, which I think you can make visible in front of the fans.*

IV1: You said this adapting in the team and changing with the communication. ***, if you compare it with the phase when you played in the championship group and then a year later: do you have a feeling that you behaved differently on the pitch? From the point of view of self-confidence: that you also are brief enough to say something or to say more. Do you have the feeling that you did something different?

*PL1: I certainly felt that way. Compared to the second championship round, the Corona championship round, I have *** but I took a position in the team that certainly went in the direction of a leadership role. When I came there, of course... *** And through the performance that I have brought, you notice that you get a certain recognition. Simply the observation phase is over, because it's clear: you're a new player and the players or everyone else waits and sees: How do I adapt to the level? How do you cope with things? How do you react to that? Are you maybe a guy who immediately seeks a confrontation if something happens? If someone says something to you, how do you take it? Because you think: “okay I don't take it personally.” I'm more like... I can handle criticism very well too and if a teammate wants to say something to me, I'm not going for confrontation and say: “How dare you criticize me? Look how you're playing.” I would never do that. No matter who it is and what it is. But rather I would try to take it in and say to myself: “Okay what now? Is he right in what he says or do I see it differently?” You can always sort it out afterwards. So, from that point of view, it was certainly the case that I was a bit more reserved at the beginning in terms of my appearance and also my standing in the team. Because I just wanted to show that quietly through work and that has been appreciated more and more and of course the self-confidence grows. It's clear, because you know what you're doing is right and is good and then you can also… which I think is quite crucial that you don't think: “okay, I'm the boss now.” But you take that and of course you think: “okay, great.” But nevertheless... It's often such a fine line between the interpersonal and the - let's say - success-oriented ambition. And of course, you also want to be successful, and you often want other people to do something for it and do as much as you do, but then you always have to... You have to find a good communicative way to say that and maybe not use it like pushing a player into the negative rather than pushing him into the positive. That was where I would have seen myself in the second championship round. That I take players with me and motivate them. And help them in this way because the team was also something different.*

IV1: Can I ask you one more question? A very simple question or maybe not. As a soccer player, do you personally believe in home advantage?

*PL1: Personally, I would have said yes. Because I just... It's just a lot of psychology. That's very clear. It's really extreme, there are some away trips and places where you just must not think about how the game's going to be, even though you know exactly how it's going to be. But that's... To accept that it is… I think as a team that all eleven players accept that, that's the difficult thing. I'm sure there are players that do that, whether we're playing in the *** somewhere or in the league somewhere away. Anyway, I think that already through the... just through the... There is the mental aspect that is advantageous, I think. Because just: it's all a little bit... First, the process is different. So, before every away game: you go to the hotel, you're there the night before, you don't sleep at home. So how do I prepare properly the night before? Do I do it the same way I do [it] at home? Probably not, because it's a different environment, you eat at a different time, you don't have - I don't know - a regular routine when to get up and things like this. And I think that this alone makes a difference, and it depends... Okay you look at things like: How is the pitch? The fact that we have a great pitch in our stadium and that it looks great, that just makes playing soccer funnier. So, you also look for this away. These are small things: Okay, what's the atmosphere like in this away game? Are you playing in front of a good [as in overtly supportive for the home team] crowd or not - that also plays a role. And the better you fade out these things, because you are just there to win the game and go home again. And if all eleven players can do that, then probably the advantage for the home team will be on a minimum or hardly there. It's simply a question of who's getting into the game better and who's going to play their match plan in the game. But I think that before the match, little things could make a small difference. I don't think there's a huge difference. And I don't think that's such a huge advantage either. But the fact that you simply adjust to certain things in the preparation and also in the mental preparation. That is - I think - what could perhaps give you [the home team] the home advantage because the team knows: okay, now the team XY is coming but we are at home, and we want to present ourselves well in front of our fans and they are often euphoric and motivated. And you have to do the same as the visiting team.*

IV1: So, you think it's more the factors before the game, but less during the game? That during the game are less factors [speaking in favor of the home advantage]: But that it's more about the feeling before the game?

*PL1: Yes I think so. I think it's like that. In the game - I'll be honest... I don't think it makes such a big difference, because the atmosphere is, seen from that point of view, like it is in any other stadium. Sure, in one better, in the other worse. But related to the game, I don't think that makes a difference. Because if you have an away game and you get a good start into the game, you don't really care whether there are 30,000 people or 300. It doesn't really matter. So, in the game I don't think I would have told a certain aspect... If so, then rather the fans… the fan side... Like okay, I would rather relate that to: in the game, if you make a good game away, the pressure is maintained throughout the whole game if the opponent… if you play at home, if you make a good game at home and maybe you lead 2-0, which is always a dangerous score… This already makes a difference than if you lead 2-0 away and maybe concede in the 80th minute the 2:1. That's different to when you concede it at home, because, of course, 80% [of the spectators] want you to concede the second one, and of course you notice that. And then it's just a matter of handling the pressure as well as possible, and the mental aspect certainly plays a role there. “Okay, I stay cool in my head and don't let myself be influenced or provoked by anything. If I play my game the same way as before the goal, then everything will be fine.” But if you have some player who loses his coolness or gets influenced or provoked, then I think that home advantage occurs, because the atmosphere can completely change and suddenly there will be another impact of emotions than in earlier periods of the game, because you were good in the game. The fans never really - because you can tell if it's just like normal support or if it's really emotionally euphoric… I would have said there's an advantage in this point. But otherwise, I would refer to the “before” as more important: How do I prepare for this game? And during the game on such things, rather like... what are such game-changing scenes. That at this point could be an advantage for the home team.*

---THANK YOU & GOODBYE---

#

#

# Player 2 (senior) transcript

IV1 = Interviewer 1, IV2 = Interviewer 2, *PL2 = Player 2*

*** = excluded parts due to possible inferences

---GREETINGS & EXPLANATIONS---

IV1: When you think back to those ghost games, what it was like without an audience, how motivated were you personally in those ghost games compared to normal games with an audience? How was that for you? Were you equally motivated, less motivated or did you notice any difference?

*PL2: I'd say that, in my feeling, I was equally motivated. Like how your approach is, or what you plan to do. Very clear. *** He [the manager] did a really good job. He kept the concentration levels really high, so that we were very focused or actually got ourselves in the right mindset more than the spectators did. Just with the communication in the dressing room, on the pitch, the players who were or are on the bench, that they are also more vocal in the game with more enthusiasm and also provide support from the outside. That's what you're missing in a game with spectators... you really don't notice that from the outside... whether it's one of your coaches or someone else shouting something.*

IV1: So you were already aware somehow, that apparently was the topic somehow, that something from the outside is missing, so to say. Some kind of stimulation. And then you also consciously... so if I understood this correctly, then you also consciously, so to speak, in the preparation before the game or just before the game or maybe also during the game, you did also consciously concentrate on the fact that you maybe push one another more, that this motivation then, this level, is the same? Did I understand that correctly?

*PL2: Yes, you just try to create the right state of mind. So that you're present right from the start, you're in the game right away, and you don't need any time to get started. From that point of view, I would say that it was very intense for us. Because normally, when you go out onto the pitch to warm up, there are already people in the stadium. There's already a bit of a tingle, a certain sound in the stadium. So it's not completely quiet, but rather you have a certain noise level and the more people there are in the stadium, the more tingling you have inside.*

IV1: And this tingling, that is, when there were no spectators, did you have the feeling that the tingling was not there as it usually is?

*PL2: No, it's not there like it usually is, no way. You try to get yourself in the right state of mind, just like we did. Otherwise, I'll say, you get it in part through the warm-up, through the fans in the stadium, and that way you have to make sure that you get it all by yourself.*

IV1: If we now briefly dive into these matches, into the league and the ghost games that you all have played so far. When you think back to these ghost games, did you perhaps somehow notice that other players, whether they were teammates or opponents, did you have the feeling that they behaved differently, that something was different about them than in normal games? Purely in terms of behavior, when you talk to a player for a second or when you talk to an opponent, talk with the referee, was there purely in terms of behavior, from the feeling that you had, something different?

*PL2: No, I wouldn't say that. Not from my point of view. Often it's just about the situations that are happening right now and then you discuss them and I would have... I don't think that would have been any different if there had been spectators there.*

IV1: Okay. Now specifically looking at the referees. We've already addressed it for a second. You said that once in a while you discuss a situation. But now specifically asking about the referees, did you have the feeling that the referees or also the linesmen or the officials, that there is something different, that they behaved somehow differently? Or do you have the feeling that it's exactly the same as with the audience?

*PL2: Well, it's not exactly the same, because of course they hear everything that we communicate with each other, or if you're standing a bit further away and got angry, then they hear it without spectators, and if there are spectators in the stadium, then they don't even hear it as much. That was different in any case for us.*

IV1: Is that something you've prepared yourself for? *** But is that something you've prepared yourself for, because you knew, okay, maybe I'm not allowed to say anything for once, because he can potentially hear it, or everything is just much easier to hear right now? Did you adjust to that or did you say "yeah, I don't really care, I don't mind"?

*PL2: Since I am not the kind of person who goes off the deep end and insults the referee, it was not a big change for me. I think the referees also understand that in certain situations in games you just get angry and upset and in most cases you can... If you talk in a normal way to a referee about situations, you also get a normal answer.*

IV1: Mmm. Now, may I ask again? You said that you are not the type of person to confront the referee. Can you think of any of your teammates who might... who might normally do that, that they might verbally attack someone, for example a referee? Have you noticed that someone is perhaps a bit reserved? Precisely from this viewpoint, because he just knew that he would be heard? Or asked differently, was this perhaps even a topic in your team meetings? ***

*PL2: No, not that. There have certainly been a few situations in some games where one or the other player has just said something out loud against a referee or about a referee, but he has then also stopped it immediately and then also immediately has gone to the player, has faced them and has then, I'd say, also warned them and that mostly helped anyway. So from that point of view, there have not been really bad confrontations. But certain players... I'd say they got truly aggressive or really aggressive out of emotion, and the referees put a stop to that relatively quickly.*

IV1: So just to summarize: Just when you think back to the ghost games, in general, was that - now compared to normal games -... was that easier, the same or harder to play those games? So now in general... away from these... whether you hear something or not, regarding the motivation... but just in general, did it make a difference for you? Did you find it easier maybe or harder or was it that you say no it was completely the same actually?

*PL2: In the game itself, of course I’d say, it was the same. The preparation for the game is much more intense now because it's more in your head and you don't have any support to get into the right state of mind and that's what the fans do in any case and you drive to the stadium and it's empty, there's nothing going on and you get into the dressing room and there are... you're used to people everywhere and there's security, there are already fans in the fan park and *** you have some traffic to the stadium. When you come down to the garage, come in and go into the dressing room, there are already some people that you see and you just can sense that. You take that... you perceive it, subconsciously. It all falls away at once. All of a sudden it's just like when you, let's say, even the training games now with spectators... you're happy when a few hundred are there. In the past you said, "Why are there so few?" Now you are happy about every spectator and that is already the preparation for the game... is already very, very intense or much more intense than with fans. And the game itself is not, because then everyone has the ambition that he wants to win at all costs and where it really strikes you is when you're cheering, I say. When you score a goal, you're already happy, but with fans it's different again.*

IV2: May I briefly get back to the preparation for the game? Did you then change something, even consciously in your routines, because you just noticed, somehow, something of the tension or something of the normal environment is missing, that you said, yes okay, now I consciously change something in myself. How you just go into the game... in the preparation for the game, you activate yourself a bit and so on. Did you do something there?

*PL2: So that I would have now added completely new things to it, no, not that. Just a bit more intense, so to speak. Just in... in the head area, in the mental area... just worked there even more intensively so that I get where I want to be.*

IV2: Can you tell us... would you like to tell us a little bit about your mental preparation, what you're doing there?

*PL2: Yes, you watch much more games, you prepare much more intensively for the opponent on... on it, watch your own scenes and engage yourself even more... with more music, work even more individually, even more in the different areas. Are just many images in the head then that you then... that you then try to collect.*

IV1: So I can just understand that this was really... was an issue for you personally, which really affected you before the game, more or less, but then in the game you were obviously really in this routine.

*PL2: Exactly.*

IV1: And then, so to speak... these external influences afterwards no longer played such a role, but really... it's really about those before... so everything that happens before the game.

*PL2: Exactly.*

IV1: Maybe in individual scenes when you celebrate or when you score a goal or something, that you notice again "okay there's something going on", but obviously, as you just told it, during the game it was really "business as usual", I'll say. Can one say that like that?

*PL2: Exactly, exactly. In the end, it was a little easier for us because the communication on the pitch was now fairer for every player. Otherwise, a teammate who is a few meters away from you might hear when you call him or when you communicate with him. Now that the stadium was empty, you could talk to the striker, you could talk to the... I could communicate well from the left side to the right side and that was all much easier and now it is again... and with spectators it's a little bit more difficult. But in the game otherwise... something like that when you're inside the action, I think you don't really notice whether there are 5,000 or 45,000 [spectators].*

IV1: Did you also change the way you trained somehow? Did you train differently or did you train in exactly the same way? So because the communication situation is different, then I could imagine that you might also... maybe then say, okay you might practice things differently, because simply now other things go easier. Was that also a topic or was it not? Did you basically train in the same way as always?

*PL2: Training was actually the same in terms of content. Our topics, our philosophy, how we want to play. From that point of view, we kept it the same. The only thing that was really... we tried to become more intensive in communication on the court.*

IV1: Yes, great. [IV2] can you think of anything else or do you have any ideas about what else you would like to ask?

IV2: Yes. And so habituation effects, so that has then also in the course of the ghost games, that with the... has that normalized a bit now, that that... or how long did it take for that to become a normal situation for you? Or, how is it now again? Are you already used to the fact that there are spectators again? Or how long do you think it will take until it's just completely normal again, without thinking about it in any way?

*PL2: I can't say exactly how many games it took until we got used to it. But it was okay, so it was already the case that we accepted it and it just went on like that. One game after the other. You prepared for it like before, just like it worked well back then and then we just went through with it and then there were just a few games last year *** where a few spectators were already in the stadium and of course you're happy about that and... but it wasn't like that, that again... So the transition from spectators to non-spectators is more difficult than the other way around. From that point of view, it's not such a big issue now that someone is somehow nervous or other things.*

IV2: Okay, yes, that's what I thought. And the only thing is that communication may be different again or more difficult.

*PL2: Exactly.*

IV2: But it's just so cool, of course, probably, right? Just, without reservations, awesome that people are in the stadium again right?

*PL2: It's great for us. *** When you take the bus to the stadium and there are already a few hundred or a few thousand [fans] in front of it. That's fun and we're really happy when we're on the pitch and the same now at home, so it's already... You enjoy it again.*

IV1: Now aside from that. So from the point of view of how it works better, but now quite honestly, what if you could choose, what would you prefer? So would you prefer to play without an audience and thereby perhaps have certain advantages, just because the communication works better or do you say I accept that now and in return I get this stimulation from outside? As I said, we are not journalists and we do not want to have an answer now somehow, which is perhaps somehow now PR adequate, but really *** now asked very, very directly, what would you personally prefer?

*PL2: With spectators in any case. So the more spectators... are in the stadium earlier, when we go out there onto the pitch, that... that's just fun. It's much more fun.*

IV2: I think it's awesome and cool that this is simply also... the fun in professional football is also there and plays an important role for the players, right? I mean, you're not machines, robots, well, you're also people who have feelings and who just want to have fun at work.

*PL2: Exactly. That... with us it's like... ***... that makes just... that's just good for everyone, if you can then also, I say, just play football, let the ball run, but before that it's just really like, where you also have to keep the intensity really high. That is for some players like okay play intensively, a lot of sprinting is not the funniest now, but someone who likes to play football, just plays around a bit with the ball, shows some skills and so on. That happens more and more in the game and then it's really fun. So you really have to do the work beforehand, really work hard, and then it's also fun afterwards.*

---THANK YOU & GOODBYE---

# Referee transcript

IV1 = Interviewer 1, IV2 = Interviewer 2, *REF = Referee*

*** = excluded parts due to possible inferences

---GREETINGS & EXPLANATIONS---

IV1: How... if you think about it now, these ghost games at that time or... well, there are none now, but the games without an audience, how motivated were you at these games, now compared to the regular games, where there is an audience? So did you notice any difference? Were you more motivated, less motivated, was it maybe the same? So in general, from your subjective point of view, how motivated were you in those ghost games compared to those regular games?

*REF: Hmm, just going through… *** It was a very strange time, because... because just during... during... well, Corona is also involved, because it's... because it was somehow... because everything else was interrupted and that [football] wasn't, that was a type of motivation that was all its own. If I really just compare now, *** then it was… I would say that the motivation to go to the game is... no the motivation is the same, the excitement is completely different. So the excitement was totally, totally different. So without spectators, it was at the latest after... at the latest, I would say, the third ghost game was like "yeah okay we'll go there". The whole procedure is much, much tighter, because you hardly have any contact with... also with the supervisor [of the referees] and so on. So we are being observed at every game. It's totally changing. You have... you go there, you don't have the possibility to eat somewhere afterwards anyway, you go in, you have a look at the pitch, you go home, you go... you do the match, take a shower and on the way home you talk to the observer on the phone. Now that's... normally that's quite different, because you're always like... that before [the match starts] the observer comes in, then you talk to all the people, functionaries and so on. You don't have all that anymore with the ghost games. You really just go in, see the players, and don't even greet them with a handshake. It's all so distant. Then yes... well, I would say it's similar in terms of motivation. *** So for me, every game is still amazing, so that... I really like to watch and it makes me happy that I can be there, at these games. So in terms of the motivation to do those matches, I don't think that anything has changed, it's just this... this excitement was different at the latest, I think, after the third ghost game. So that you really have this, this tension, excitement.... It was a completely different level. So when I go into the game now, for example, and think back to situations where you know exactly how he's going to fall [to the ground of the pitch when being fouled] or so on, the whole stadium would normally be screaming. And that just doesn't happen in the ghost games. Something happens, someone shouts "hey" and you think to yourself "oh, please, I can't give that now". And normally I think maybe I would have whistled a few things differently, if I knew exactly, okay now you have the whole stadium involved, then he also remains lying there longer, because then it's not too stupid for him, because he knows he has the support of the whole audience. Yes, the motivation then... no the motivation I would really rather say the same for my... for my enjoyment, motivation like I... how I look forward to the BL1 [first division] games and approach a game. I would describe those very much the same, yeah.*

IV1: You said before that the excitement has become a little less after the third game, so to speak.

*REF: Mhm.*

IV1: Is it now, so to speak... so do I understand that correctly, that the excitement is now generally, that it is different now, less or now the excitement, because you just exactly this... just no longer have that audience, so that you just know it's a private event. So which of the two, so to speak - now considered in terms of excitement - has it now become less, because it was now just so to speak somehow new and you just did not know what was coming and therefore the excitement [went] down? Or was it now the case that this excitement, this tingling, because there's just a lot going on and so on or was that the... this excitement, what you mean by that?

*REF: The excitement that has diminished because... was that.... because the ghost game issue was simply a new one.*

IV1: Mhm.

*REF: And we had these certain topics, that you hear everything we say as referees. Or even - actually worse for me - what the... what the players all say to us and we actually always go over it like this, because we know anyway that this is normal and that this is also okay, because I can't imagine that there is someone who has something against me as a person. But this thing, to find the right balance, when you then look at it on TV afterwards and think "dude you really hear everything they said to us" and then they [the media reporters] ask why we don't deal harder with them. This justification you have to do then why you don't book them all off. That's more of this exciting issue because it's just a whole different environment then. Now with the spectators... it's just... it's like a, I don't know, when you go out there and you have a bunch of spectators that are loud, then you just really realize that it's like show... it's show and soccer is just.... I think you need... I took a little while until I could accept that this is simply some kind of circus. So you march in there and then we all join in because it's just the business. And then you have to... and then it's no longer just football, no longer just making decisions and everything like that, but no, it's a circus. That's the other excitement, that is, at the ghost games, yes, what should I do there, if I... it's enough if I somehow look at him angrily or at least pretend that I look at him angrily, then he's already quiet. I don't need much. Only if the audience is also watching, then you have to make some kind of gestures and... that everyone knows that you've said something to him now.*

IV1: Mhm.

*REF: So that’s missing, yes.*

IV1: Maybe I'll just interject here, because it somehow leads over to the next question perfectly. Is this something where you would say that - you've actually already answered it a bit, but I'll ask again specifically - do you have the feeling that you behaved differently during the ghost games? Have you changed your behavior in some way?

*REF: Yes, I think... so it's more suited to my nature, because I don't like making that many gestures and big... so I like this... so the... the task, the role of the referee, to fool around so much and and to appear so big for the audience and so on, I had to learn that first. So for me, I got from my feeling the... the... the gathered game management or the respect of the players always just through decisions. So when they saw, okay well I understand what I'm doing, then they were mostly all calm. And you can do that in a calm tone in ghost games. Now on Friday I was... *** I have now held a match and then it was really loud again since a long time. And then I noticed how I had to shout again with the players and how I had to yell at them. That's when I noticed how exhausting it is to have such an interlude all the time with just a laugh, so that they don't think I have to talk to them like that all the time... that I have to talk to them so harshly. And in the ghost games you really... you were able to use all the nuances of the language. So... it was enough if you said a loud "Hey" for a moment and then immediately stepped back and talked to him normally. Then... then a player hardly yells back at you if you don't yell at him. But you have to yell at him now because it's so loud. It's just, yeah, funny.*

IV1: No [it] is... So super interesting, really. So perfect. You've already briefly mentioned the games…

IV2: Can I just... Can I just interject one more time? And that is, do you have the feeling that you are now actually not only interacting with the players, but also to a certain extent with the audience? You've described that a bit now, haven't you? That you also have to somehow bring your decision closer to the audience, so to speak? Is that also part of it?

*REF: Yes, that... so... that's something that... so in my nature just not so... so... so... I had to learn it, let's put it that way. Well, I was a soccer player and I have always understood why someone gets upset at the referee, because I know that it is all... that it is very difficult to understand and I also always complained about the referees. Until I then just... I've held two or three matches myself, then I've been much calmer on the pitch and yes. But that's just, as I said, this understanding that especially in the highest league it's just a show. And that if you look at the Champions League and everything else, that these are just perfect showmen who - no matter how good the decisions are there - but you have to get where they are and not just in front of 60,000 in the stadium, but they have an audience of millions and they have to sell it in a good way not only in the stadium, but also on television. And this... this interaction with television, even if you don't want it, but that's simply my job. That... you become much more aware of that with an audience than in the ghost games. That there is simply someone who is watching.*

IV2: Okay, thanks.

*REF: There definitely is a difference.*

IV1: You briefly mentioned the matches before. Because... so now we talked about the referees, talked about all of you, talked about you, that maybe you all or you maybe.... someone behaved differently. Do you have the feeling that the players behaved somehow differently, that there was something different in these ghost games? Or do you know maybe, I mean you know players also like it was before, you know maybe some guys... maybe where you just know, okay he's like that maybe, he's like that sometimes. Did you maybe notice something or did you have the feeling that they behaved somehow differently without an audience?

*REF: I think you have to separate that a bit. I'm trying to sort out the impressions. At the beginning, in the first few games, I had the feeling - it was Corona-related - that everyone was happy that they could play football again. They were all rather reserved, I had the feeling. Everybody didn't want to... there was also this whole hygiene thing where they didn't approach each other. So... but after some time I had the feeling... how was it... we had a short phase where there were spectators again last year in August and then it was again... then lockdown hit again anyway and then again without spectators. I then have the feeling that it was actually relatively normal again, so "normal" (shows quotation marks). Maybe... so from the judgement of fouls I felt they.... they try less so... so to draw quite cheap fouls. So I have had the feeling that just with this lying down and this screaming around, which you otherwise maybe happen to see in the lower leagues... what's easy. That has then also become a bit uncomfortable for them, I think. So from my feeling, because if you don't hear anything and only the stadium microphones and then.... so I think for my feeling that they have then already backed away a bit from the communication and from that very cheap foul drawing. So now only [judging] by the match evaluation.*

IV1: Mhm.

*REF: Yes, otherwise… so I had the feeling that they then just now when it's not loud in the audience, that they just then also talk to me more quietly. And that this is simply a completely different... is a different basis for a conversation than if they simply forcedly have to look at you because it is loud. But those are the main...*

IV1: So you say somehow that already this... these... these acoustics simply were so maybe a bit of the main or that... that's how I understood it, if I subsume it now a bit so what you have said so far, I have the feeling that actually so again and again these acoustics... so you always address these... these acoustics. Not that you can maybe... because you understand each other better, because the communication works better, because maybe you hear more, which you might not hear otherwise.

*REF: Yes.*

IV1: Is that so maybe somehow... up to now maybe the quintessence from this whole ghost game thing? That simply the acoustics are different?

*REF: On... For me is a... Definitely a big point already. Because even now if you just... so that doesn't... doesn't need a crowd, but if you have 500 people who shout or drum and just let the players... or even that a referee gets a certain response for his... for his actions on the field. So as soon as I whistle, the spectators give you something or they don't give you anything. It depends in whose favor [the dicision was made]. But if the players realize that they... that is my interpretation... if they in their... in their appearance experience a resonance somewhere in the stadium then... then... then something happens and I believe that this occurs most likely through the acoustics, yes.*

IV1: Okay. Now, just in retrospect, when you think back to all the ghost games, that is, what you experienced there. Was it easier for you to work, I'll call it that, yes, or to do your job, was it the same or was it even somehow more difficult? So there are probably several arguments that you have to sort out.

*REF: Mhm.*

IV1: But now, if you just try to break it down like that and just evaluate it like that, how would you.... how would you say that?

*REF: I think for the... for the big games, when really big names collided, it was easier. For me. Well, I held two or three really important matches. *** And in both matches I was glad afterwards that I could really concentrate one hundred percent on the task. So that I could just really stay totally focused without somehow having an additional factor from the spectators. On the easier ones... "easier" (shows quotes)... what's easy? The easier games are mostly... it is just so also... when they are... are smaller clubs, which is with less spectator potential and less media interest, and if it's not directly about... about any UEFA spots or about a relegation. In principle, these are easier games, because the decisions are not as valuable afterwards. So it's not about that much. So of course it's about enough, but just... you're not immediately in the newspaper if you do this or that. So for the easier games, I think I prefer the audience, because if the player is just going back and forth and you have to keep the tension... you're happy anyway, if you can slow down for a moment during the match somewhere. But if you then... if nobody pushes you anymore, because the audience doesn't scream either, and then you think to yourself "yes, he can't get the ball anymore anyway, I don't need to run after him now", because then you maybe don't go into the counterattack with... with the same speed and you are missing that then. If he still gets the ball and you're suddenly 25 meters further away instead of 25... instead of 15 meters, then you just don't feel so comfortable with the decision anymore.*

IV1: Mhm.

*REF: That means that you don't have the audience to boost you, so that you always know exactly what it's all about today. Every BL1 [first division] match there's enough at stake.*

IV1: Mhm.

*REF: Yes, it is like that.*

IV1: Mhm. I understand a little bit that maybe the meaning becomes a little bit less when the audience... so if the audience is not... so, so now, that the meaning becomes less, if there is no audience. I can pick that out a little bit now.

*REF: Mhm.*

IV1: Can that be... or is that now my interpretation... or is that already something that you somehow also feel? I mean, you said earlier that you are equally motivated and stuff and it's all kind of like that....

*REF: Yes.*

IV1: Now it’s more fun.

*REF: Yes.*

IV1: But is it perhaps a little bit like that, that this meaning of this event, as you also said, then simply also becomes a bit less now maybe, if simply then that somehow takes place in some kind of vacuum?

*REF: So I think even though I certainly don't want to admit it like that now, that it is like that in my head. It simply gets less meaningful then. So that reminds you more like when you just hold a Landesliga [fourth division] or some similar match then, where there are just fewer spectators or otherwise somehow. This... this spectator thing... that does something to you. It doesn't matter if you're going somewhere internationally and suddenly there's a stadium that has a certain look and no people are coming. That certainly does something and when I think about whether that messes with my motivation when I'm there. Maybe there's something to it. When you are there in this... in this... the question is whether I don't have to rethink that with the motivation. If it's really about these feelings that I feel there, when I go into the stadium and... and... and you hear the ball bounce.*

IV1: Mhm.

*REF: So I would be interested in what I would say in 10 years, if I had really held so many matches that I just, as I said, not every game is just awesome for me that I am allowed to hold it. Would be an interesting hypothesis, I actually can't really.... for me it's just really like that, when I... I'm happy every time I get a text message that I can hold a match this weekend. So...*

IV1: It's, it's just also totally.... now really a subjective perception. So it can also be that...

*REF: Yes.*

IV1: Now everything is... it's not our demand that we somehow receive the one hundred percent truth from you now.

*REF: Yes, I'm trying to incorporate as many factors as possible that resonate there now.*

IV1: Yes.

*REF: But it's... the meaning is just different then when you get to it and there's nothing going on.*

IV1: I would just like to ask you a quick question about your referee colleagues. You are... you are not alone at the games, normally there are four of you. Have you somehow, now apart from maybe that maybe the communication is getting better, so we've already kind of heard that now, because it's simply also easier to communicate.

*REF: Yes.*

IV1: Although you all use headsets and yet it may still be easier. But did you maybe kind of notice something with your colleagues? Did you notice anything about the cast? So with those who have led the game with you there, that they have behaved somehow differently? So not yourself, but maybe also your colleagues? Did you maybe notice something that was different?

*REF: Yes, so mainly when I think about communication on the pitch... so maybe what's always important to me personally... is that... that I always have the feeling that everyone is [mentally] present. And when everything is quiet all around, then you can hear everything. And if it's just quiet everywhere, even on the radio, then you might think that they're not really there, the assistants or something. Because that's simply as a referee and assistant... you feel the game quite differently. So when you're standing on the line outside, you're usually calmer. You don't have to talk to the players, because the players have less respect for an assistant than for the referee, because the referee books them and the referee gives the penalty and not the assistant. And therefore you are always calmer as an assistant. If everything is already calm, then... then the referee must really, really trust the assistant, that he is fully involved at all times, no matter whether he has a few thousand spectators on his back or not. Who really want every decision to be right. And not that he just thinks, "Well, I'll give it that way, because it'll fit." But that in retrospect ... maybe I felt it somewhere during the games that I sometimes felt less comfortable because I didn't have that feeling of... trust in my assistants. There are just some where you know, the.... they... they have a very similar understanding of your approach, how you officiate games and what you need as a referee. Sometimes that helps when you push each other just to be able to... yes, to finish the match in a good way. So trust is certainly the most important thing amongst each other. And because that is rather... yes... I think that sometimes people make more of an effort, including me, when there's more at stake, when there's more pressure from the outside.*

IV2: What I would still be interested in, following on from that, is that you say "okay, confidence in your assistants is important and if... if the audience is there, then you can assume that they'll make more of an effort anyway", that kind of thing. To what extent did the ghost games influence your self-confidence? That was also one of the questions we asked in the questionnaire, and your colleagues gave very different answers. Could you perhaps elaborate on that a bit more? On the self-confidence or the security in your... in your decisions?

*REF: Yes, for me it's a very... It's a really exciting phase for me right now. For... for... *** just in the time of the ghost games, actually a short time before [the ghost games], in a phase... I got into a phase where I had the feeling "I can do this" and I've just arrived there now. And that almost increased with every game. So, the ghost games were helpful for me because I was able to concentrate even more on making these... these decisions. What... what is a foul, what is not a foul. And those... those... So, all the strengths that I have in that way, to make full use of them. Of course, in contrast other things fall away, because... because the... I would rather put my weaknesses into a different perspective, rather to interact with the audience. With the... with the, yes, to “sell” everything [i.e. every decision] and “to do around” [i.e. create a show]. And that just falls away there. So, for me it was just such a phase that helped me, and I could get even more of a good reputation, I think, without an audience. And for me that was, yes, rather helpful. Because I just got more self-confident, in the sense that I know that I can do it.*

IV2: Mhm.

*REF: I assume that the process would have been slower if... if there hadn't been any ghost games. But I was definitely already in a phase where it was getting easier and easier, because I had put that first year behind me. Yeah, well, I'm... I definitely... I found it easier in terms of self-confidence.*

IV2: Is that sometimes also the case, that it's through the audience... that you get a confirmation in your decision or that it can also give you hints or is that not the case at all?

*REF: Oh yeah. So, some things... Let's put it this way. An example: If someone goes to the ground easily [after a tackle or foul] and you think to yourself "he just wanted that [foul] now" and if there are many [people] screaming at the same time, then it's easier to whistle. Yes, so even if it's... there are 50/50 fouls where you say, "Yeah never mind." But if it's already 60/40 [or] 70/30, then you should actually always whistle the fouls, because a foul is a foul. But if it's really like that... Sometimes it makes it easier to accept these fouls... for me to accept them, because every audience, if everyone sees that, then the defender is the one to blame. So that can make some decisions easier, because you think to yourself: "Well, others see that, too, that you [the defender] were just clumsy." Regardless of whether the other person wants that. So, it makes some decisions easier for me if I know that it just looks like that on the field.*

IV2: Okay and that affects mainly the home team, doesn’t it? From the other [away] side, the audience will not go along like this?

*REF: Yes, of course. But even if it looks like a foul... Well, that's really the... Well, I think the soccer consumer... There are very different opinions about what is really a foul and what is not. So, these experts, when I say... So, experts from the football players themselves. So, if someone has played from the regional league upwards, then I have the feeling that they can assess what really was a foul and how he wanted [to do] it and what not. But regarding the rest of the audience, these are TV consumers. Yes, well, every shirt-grabbing is a foul and must be yellow. These are just very different consumers, and they are all sitting in the audience. Sometimes more of them [experts] and sometimes more of them [TV consumers]. So, whether that's necessarily the home audience... But yes, of course, rather the home audience.*

IV2: I mean... What we also found in our conversations with the players or what emerged: First of all, that there are total differences in the experience of the players, and for one it had a different effect than for the other. But it is also a factor, that you as a player use the audience or also the impulse of the audience, which the audience gives, when you realize there is a 50/50 decision and the referee is also not quite sure, because it was not a clear situation... that the player also consciously takes the audience with him and makes a bit of a fuss again. We talked before about “show and circus” [of the players] and so on. To what extent do you think, or have you been able to observe that this has changed. You already said: the obvious things [fouls or tackles] didn't happen to you [occur in your matches], but is that also a factor that you always have in mind or how has that changed?

*REF: Well, I think that's just, as I said before... Well, they've tried it a lot more cleverly. So, with 50/50 things or less, they did not even try anymore. In 70/30 [situations] far more again, but I have also had the feeling that they have calmed down much faster again. If you say, "Okay good, you didn't whistle." Okay no, it was not that easy, but that one remains quite long in the penalty area, just to provoke.... It was in the games in which I was referee... I cannot remember that anyone for a long... wanted to achieve something without an audience.*

IV2: Our studies were a lot about emotional behavior towards provocation and so on. Has that... I mean, how about you with [your] emotions as a referee? Are you someone who shows emotions and has that changed now? So, has that changed because of the ghost games? Or do you say that it doesn't really matter to you personally now... So, from the emotional [point of view].

*REF: I don't know, I'm probably not a typical example. Or typically, no... but there are others, but you can hardly... well, that I get emotional... I have to think about when I'm really on the field... that something annoys me afterwards, that was unfair, that... I think about it for a long time [after the games]. But on the field I... well, I'm always told that I have too much understanding for everything and put up with too much, because... well, really, I don't care so much when someone runs to me... It just matters a lot for them, for them it's really about every bonus, about every game. Maybe a scout is watching them, then... I don't know. Or someone is substituted and finally gets some time to play. For them, every game is about a lot. And when someone yells at me, I still feel... I don't know... I always must overcome myself and think, okay well, I can't let myself be yelled at like that. I mean, I realize anyway, when everyone comes, then I give back, because that's not possible. But so... so from the emotions, I was already calm before and I think during the ghost games probably even calmer, but that is... the difference is not that big.*

IV2: So, like you said before, it's more of a personality concession anyway.

*REF: Exactly so my characteristics yes are... So, you have to provoke me really long.*

IV2: Well, I mean, that's certainly a good attitude from a psycho-hygienic point of view, because you also mentioned that before. You don't assume that someone has something against you personally, but…

*REF: Yeah, exactly so it…*

IV2: So, you can separate [it] well from you as a person.

*REF: Yes. For me... Well, it helps me. Sometimes it's just maybe... Well, it's not always good when the referee... some emotions you just must play like, but that works well. So, if you say: "Woah..." I can't talk like that [here], but if someone really annoys me, then I have to express it in a way that he believes me. Because otherwise he just comes on to me all the time. But that it really irritates me, that rarely happens.*

IV2: Okay. I have one more question about the progression. How did that change as well? I mean, we have... So, it was like this: from March onwards there were ghost games, from August onwards there were again games with reduced home audiences allowed, and then there was lockdown again and then…

*REF: Mhm.*

IV2: Exactly. And the 20/21 season completely with ghost games.

*REF: Mhm.*

IV2: How has that changed for you? Or did it change? Or did it lead to habituation... So, was it a habituation effect or did it normalize or how did you... was there a dynamic inside the experience?

*REF: Well. It's very hard to separate that from my general feeling, from the whole corona ups-and-downs. So how much that resonates there. Um... I'm trying to go through it chronologically: I think it was May 1st or something... just before that, where we were told... or a week before May 1st, or was it the 31st? Anyway, or no, first of May, sorry. That's when I got a text message that it wasn't going on, that in any case the clubs were saying, "we don't have a season, it's over now and we'll wait until July/August." I can remember that I actually... because before that [moment] we trained into the blue. So, everybody with their possibilities... always running around with the Polar-watch and we never knew, does it go on or does it not go on. And [we were] totally unsure. And also, everything only in individual training, without contact to any of the colleagues.* **** So you stay in contact and I was always... So, *** you can hardly train, because I think the fitness center was closed at that time, everything was closed. You don't have a treadmill... And so on. I used to go running. *** That was the status before [the start]. Then, a week before May 1st, I think it was... we got the message: "It's not going to be played anymore." I can remember then, I went to celebrate somewhere properly, where it was possible [to celebrate]. And a few days later we got: "Ttraining starts and in 3 weeks the games start again." Something like that. So that's how it is in my head. But it can also be that I'm wrong by a few weeks. So, it was like: “Puh how do I manage now to get to this state of fitness without regular training?” Also on the lawn... it's just... so I'm normally... so I do my job very intensively because I always go hard, and I'm knackered after that... after a match. And then also to referee during the summertime, that was... without a proper training I often thought to myself: "Phew, how does that work?" Then we have always refereed two games per week, so it was very intense during this time. If I from the dynamics ... There were just so many games, I was then again glad that we had a short break. Whereas the break was, I think, not too long and then it went on again. So, from the dynamics, it is mainly so... my mood was a bit Corona-dependent. So depending on how happy I was about it or how much I was annoyed by it, the general situation... of course, knowing that you're privileged to be allowed to continue this whole “circus” at all, that I can pursue this job, while others in my area are on short-time work [= a concept that reduced work hours in order to fight the corona virus] and it still annoyed me that, well, I think I can already remember that it's all... yes, of less value when it's in an empty stadium. Yes. It was a strange dynamic. I don't think I felt it was positive. So, when it has settled in a bit and you think to yourself: "well, it's always going to be like that now." But it's hard for me to separate that from my general state of mind during corona. So, when you always hear: "now it goes on, now we have lockdown, now we have this and how this is... Yes, we just play without spectators and so on." Then it's going to be like that again, because it's a weird... you go there, and you go back home. So that's with the ghost games... it's like that. You go there together by car, referee, hardly talk to anyone because no one is allowed to go into your area. No one is allowed to be in the referee's booth and so on. So, there's this... the whole flair around, where you can chat with someone from time to time or something... you just don't have that anymore. You are more alone, as the referees are mostly, because hardly anyone comes into contact anywhere. Normally, you have this. So, in most cases you get something to eat and you can sit down somewhere in the area of the VIP room and you come to have conversations, if someone wants to talk to you and that, which is often enough... Okay, those are rather, when time goes by after the game, then everyone is normal. Now you don't have that anymore, you just go back home and think to yourself: "yes, it is over,[on] to the next one."*

IV2: How is the situation for you now? Is it now a normal situation again or a strange mixed situation? Or how...? So how is the situation now with the reduced home audience, but still with all the corona-measures? So, does it feel like a normal match day again - assuming you can still remember what normal feels like - or is it still a ghost game atmosphere?

*REF: Um... it's not normal yet – I think. But it's already far different. So as soon as they boo you, if you decide anything against them, you already know it's normal. Yes, but it is still relatively empty [in the] stadiums. *** It's much more normal, but it's not yet... so a stadium where all the stands are full or all the stands are occupied, that makes a difference than when you run into a corner and there's no one. So even if I now really think about it. *** It was only afterwards, when I saw the TV recording, that I was surprised how empty the stadium actually was. Because during the game you hardly have the opportunity to look at the audience. You're always on the field, you're always... looking to see if someone – does not matter where the ball is - if everything is fair. It was pretty loud there, because there were quite a lot of *** away fans who made a noise of fuss and after the recordings I thought to myself: "the stadium was actually almost empty." So, from that point of view... it was almost more normal, where... although there was no one there... so only a few.*

IV2: But that's interesting, that the background noise is also... such a factor. You can also see that in the studies.

*REF: Yes, more than the visual things, I would say. I don't know, maybe when they set off “Bengalos” [pyrotechnical tools] somewhere or something. But it has only happened to me very rarely, but the acoustics are certainly different, yes. Also, the other way around, if I now... that wasn't a ghost game, but likewise... *** there was no one there. They played against someone, I don't know anymore. But you just hear... The ball hits the ground, and it reverberates, when it hits the pitch, it reverberates under the roof of the stadium and when you hear that, you think to yourself: "Wow, I'm playing at my house or somewhere." So, the acoustics make a lot of difference, yes.*

IV1: The acoustics is so far where most of the studies hack in. So, what I have now in my head is that most studies that deal with the home advantage or with this bias, with this home advance bias… There actually... most of them talk about it being related to acoustics. Whether it's affecting you… on the other side maybe to some degree or just also that it changes communication. So, acoustics is apparently already a very big component in soccer.

*REF: That means more than the number of spectators?*

IV1: Yes.

*REF: [If they are] smaller stadiums that are just louder.*

IV1: Exactly, yes, so for example... there are, for example, studies that have looked at, for example, whether the home field advantage changes since there is a running track between the audience and the playing field. This means that you are... you are simply closer. Yes, this changes the acoustics again, it's more intense. So that should somehow be... somehow related to that.

*REF: I can certainly confirm that. The runway is a 100:1 relation.*

IV1: That is, LASK has a worse home advantage or not LASK, [but] Blau-Weiß Linz or so for example in the “Gugl” for example.

*REF: Exactly yes. That was always... also Happel Stadium.*

IV1: Yes, or Happel Stadium.

*REF: I think in Kapfenberg there is also a runway.*

IV1: Exactly Kapfenberg.

*REF: But that's really... Refereeing there, that's also not... I don't like that either. So that's... yes that's right actually. It just makes... It also takes away... Yeah, you'd probably have to define the meaning of motivation in my head exactly, like you asked me at the beginning. It's just a very different feeling to go out there. That's... yeah, that does something.*

IV1: May I ask you something else, just out of interest. So, it doesn't quite fit in 100%, but in terms of acoustics. Do you think that helps you, that you can hear fouls, for example. So, if you for example... when the players run into each other. Or if, for example, something... You hear when a foul happens and if there are now, I don't know, 30,000 people in the stadium, you don't actually hear that, do you? Or do you still hear it on the field when they converge. So, I could imagine... my hypothesis now would be that actually because of the acoustic information, like shin to shin or somehow the shoe crashes the shin cap... that should help that you can evaluate fouls better.

*REF: Yeah, so specifically with shoe to shoe... shoe to shoe is a classic one.*

IV1: A classic one. Yeah, exactly.

*REF: Well, that helps a lot, of course. So, you can... sometimes you can still hear it, I think. Sometimes you really ask yourself why you heard it, especially because you only have... you only have one ear. The other one is completely closed. It's already happened to me that *** I didn't notice... that there were two people standing there. Because you just don't hear anything here and you only hear there. And yes, this acoustic aid for fouls or for the assistants is actually the most important thing. So, the assistants have the microphone on the right so that they can always hear the ball or the ball play on the left when they're offside.*

IV1: Ah, okay.

*REF: And you can hear that very well. So, when he hits the ball with his studs. And the ball is usually well pumped up in the Bundesliga, you hear that wonderfully. You don't even have to look at it anymore. For the assistants it's always stressy, because they must have a pendulum look all the time and if you can only concentrate on the "clack", it's for the assistants... my hypothesis is, I think, even a greater effect of the ghost games, that they just always heard that when the ball was hit.*

IV1: Now one could look at whether the offside decisions were correct more often in the ghost games than in the regular games, right?

*REF: Yes.*

IV1: So that's... that's nothing what I was thinking about, that the [noise of the] pass is a cue for the assistant. I've always thought of fouls. I am playing in a low division and there you always hear it very well. So especially in the penalty area and stuff... I often think to myself when I hear it, "oh god, hopefully he [the referee] didn't hear that." Because that was like, you know... But that... I haven't really thought about that yet, that in the moment of play, that's actually exactly the cue that you need to see where he's standing right now. That would be really interesting.

*REF: Yes, there would be the idea of how to... It's always a question... some... the problem with an offside decision is: You often can't resolve it properly even in the Austrian Bundesliga. I drew lines again just yesterday. Even with our cameras... we have now... yesterday we had: 6 real cameras. And that's not bad... in Hartberg, for example, you can hardly resolve offside because the cameras are so bad. So... But my hypothesis, which I can definitely underline, is that it [ghost games] definitely made it easier. Yes, I definitely believe that. Whether they were really better…*

IV1: Whether that was more correct?

*REF: Yes, whether they were more correct.*

IV1: We'll have to look at that. We have to... our study assistants have to do that.

*REF: Yeah, good plan.*

IV1: Yeah, that's tedious. No, just kidding, but definitely interesting.

---THANK YOU & GOODBYE---
